# Concise total synthesis of two marine natural nucleosides: trachycladines A and B

**DOI:** 10.3762/bjoc.10.176

**Published:** 2014-07-24

**Authors:** Haixin Ding, Wei Li, Zhizhong Ruan, Ruchun Yang, Zhijie Mao, Qiang Xiao, Jun Wu

**Affiliations:** 1 Jiangxi Key Laboratory of Organic Chemistry, Jiangxi Science & Technology Normal University, Nanchang 330013, China; 2 Marine Drugs Research Center, College of Pharmacy,Jinan University, Guangzhou 510632, China

**Keywords:** marine nucleosides, natural products, total synthesis, trachycladines A and B, Vorbrüggen glycosylation

## Abstract

We report the first total synthesis of trachycladines A (10 steps, 34.2% overall yield) and B (11 steps, 35.0% overall yield) by using 5-deoxy-1,2,3-tri-O-acetyl-β-D-ribofuranose as the starting material. The critical step was the SnCl_4_ assisted regio- and steroselective deprotection of perbenzylated 1-*O*-methyl-5-deoxyribofuranose. The enzyme adenylate deaminase (EC 3.5.4.6) was successfully applied to the chemoenzymatic synthesis of trachycladines B.

## Introduction

Marine organisms are a main source for the great diversity of naturally occurring nucleosides [1], which play an important role in medical research and pharmaceutical development, especially for the clinical treatment against malignant proliferation and infections by fungi, bacteria and viruses [2–7]. The initial discovery of marine nucleosides can be traced back to the identifications of spongothymidine and spongouridine in the early 1950s from the Caribbean sponge *Tethyacrypta* [8–9], which subsqeuently led to the commercialization of arabinofuranosylcytosine (Ara-C) [10], arabinofuranosyladenine (Ara-A) [11], and azidothymidine (AZT) [12] for clinical use.

**Figure 1:**
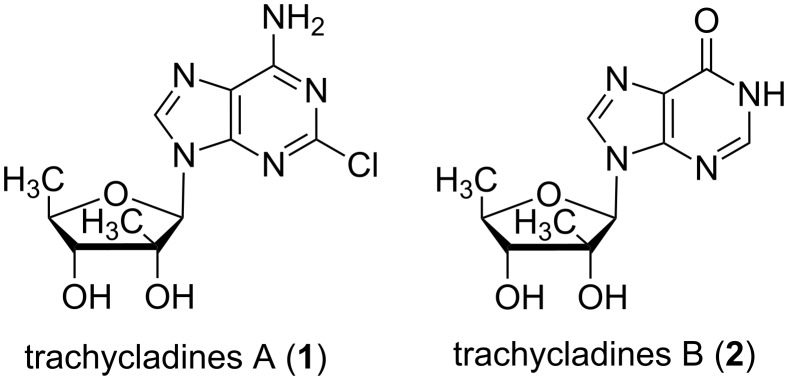
Structures of trachycladine A and B.

As a key class of marine natural nucleosides, trachycladines A and B (Figure 1) hold an important position in drug development. Both trachycladines A and B contain a rare naturally occurring 2′-*C*-methyl-5′-deoxy-D-ribose sugar moiety. The biological activity of trachycladine B has not been reported yet due to its insufficient availability in natural resources. Trachycladines A, which was isolated from the same sponge natural product *Trachycladus laevispirulifer* (and also from the sponges *Theonella cupola* and *Theonella* sp) in 1995 [13–14], exhibits significant cytotoxicity in vitro against several human cell lines such as leukaemia (CCRF-CEM, IC50 0.4 µg/mL), colon tumour (HCT-116, IC50 0.9 µg/mL), and breast tumour cells (MCF-7, IC50 0.2 µg/mL) [13]. Furthermore, such 2′-*C*-branched ribonucleosides are also potential agonists for adenosine kinase receptors and play an important role in related drug discovery [15].

To facilitate the discovery of lead compounds as anticancer reagents from marine nucleosides [16–18], the total synthesis of trachycladines A and trachycladines B are reported herein allowing to assemble their unique chemical structure for the first time.

## Results and Discussion

Until now, there is only one publication about the total synthesis of analogues of trachycladines A and B. In 2005, Enders et al. reported the first asymmetric synthesis of 4′-*epi*-trachycladines A and B in 14 steps. They used 2,2-dimethyl-1,3-dioxan-5-one as a starting material and employed the SAMP-/RAMP-hydrazone methodology to obtain an overall yield of 18–21% [19–20]. Due to the formation of the opposite configuration at C-4′ and a lengthy synthetic route this strategy is not suitable for the total synthesis of trachycladine A and B.

**Figure 2:**
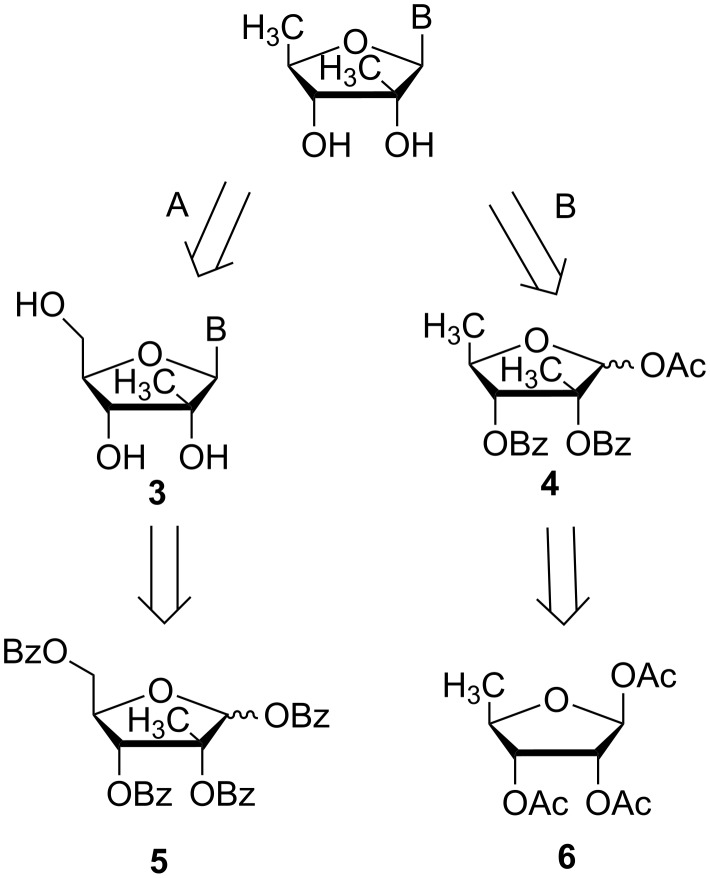
Retrosynthetic analysis of trachycladines A and B.

From a synthetic point of view, the target nucleosides could be synthesized from either 1,2,3,5-tetra-*O*-benzoyl-2-*C*-β-methyl-D-ribofuranose (**5**) (route A) or 5-deoxy-1,2,3-tri-*O*-acetyl-β-D-ribofuranose (**6**) (route B). The corresponding retrosynthetic analysis is shown in Figure 2.

In synthetic route (A), nucleoside **3** could be synthesized by using 1,2,3,5-tetra-*O*-benzoyl-2-*C*-methyl-D-ribofuranose (**5**) as a carbohydrate acceptor by a Vorbrüggen glycosylation with the corresponding silylated nucleobases and a Lewis acid as a catalyst. As the key intermediate for the preparation of the anti-HCV nucleoside valopicitabine (NM-283) [21–22] and related nucleosides, there are suitable methodologies for the synthesis of carbohydrate **5** [23]. After the selective deoxygenation of the C-5′ hydroxy group of nucleosides **3**, trachycladine A and B could be afforded. According to the preliminary results from our lab (unpublished results), the deoxygenation procedure of the C-5′ hydroxy group was accompanied by several undesired side reactions.

Then we turned to synthetic route (B), which utilizes carbohydrate **4** as a Vorbrüggen glycosylation donor. Firstly, without the deoxygenation of the C-5′ hydroxy group at the late synthetic stage, carbohydrate **4** is a versatile intermediate for the diversity-oriented synthesis of the related 2′-*C*-β-methyl-5′-deoxyribonucleosides. As the key intermediate for the preparation of the antitumor drug capecitabine [24–25], 5-deoxy-1,2,3-tri-*O*-acetyl-β-D-ribofuranose (**6**) is commercially available with a cheap market price. In addition, the regioselective cleavage of the 2-*O*-benzyl protection group of perbenzylated 1-*O*-methyl ribofuranoside by using SnCl_4_ has been widely used for the synthesis of 2-*O*-substituted nucleosides [26–30]. To the best of our knowledge, an application for 5-deoxyribofuranoside is not published. This step would be vital for the introduction of the corresponding 2′-*C*-β-methyl group in our synthetic strategy.

**Scheme 1:**
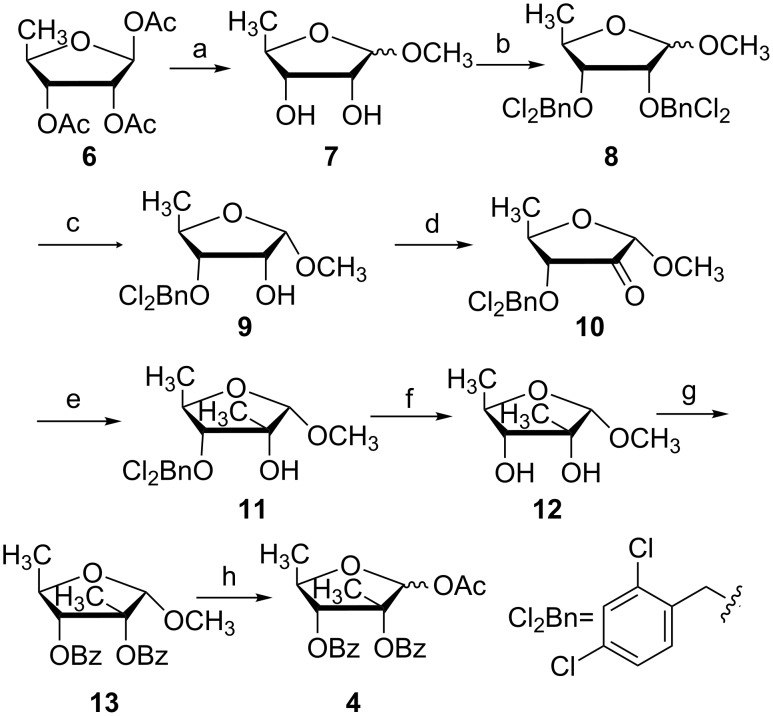
Synthesis of 5-deoxy-1-O-acetyl-2,3-di-O-benzoyl-2-C-β-methyl-D-ribofuranose (**4**). Reagents and conditions: (a) i. CH_3_ONa (cat.), CH_3_OH, 0 °C; ii. Dowex-50 H^+^ resin, pH 5, rt, overall 93% yield in two steps; (b) NaH, 2,4-dichlorobenzyl chloride, DMF, 0 °C, 95%; (c) SnCl_4_, DCM, 0 °C, 87%; (d) Dess–Martin oxidation, DCM, reflux, 92%; (e) CH_3_MgBr, ether, −10 °C, 87%; (f) H_2_, 20% Pd(OH)_2_/C, Et_3_N, THF/EtOAc 1:1, rt, 91%; (g) Benzoyl chloride, DMAP (2 equiv), DCM, rt, 92%; (h) Ac_2_O/AcOH 1:1, H_2_SO_4_, rt, 84%.

Our synthetic route to 5-deoxy-1-*O*-acetyl-2,3-di-*O*-benzoyl-2-*C*-β-methyl-D-ribofuranose (**4**) is shown in Scheme 1. Treatment of 5-deoxy-1,2,3-tri-*O*-acetyl-β-D-ribofuranose (**6**) with catalytic NaOMe in MeOH gave 5-deoxy-D-ribose in quantitative yield. Without further work-up, an excess of freshly prepared Dowex-50 H^+^ resin was added to the mixture until pH 5 was reached. After stirred overnight, 5-deoxy-1-*O*-methylribofuranose **7** was obtained as a mixture of anomers (α:β = 2:3, 93% for two steps). Then, the other two hydroxy groups were protected as 2,4-dichlorobenzyl ether. Compound **8** was isolated in 95% yield after reacting compound **7** with NaH and 2,4-dichlorobenzyl chloride in anhydrous DMF. 2,4-Dichlorobenzyl chloride was used because it is less irritant than the usually used benzyl bromide.

**Figure 3:**
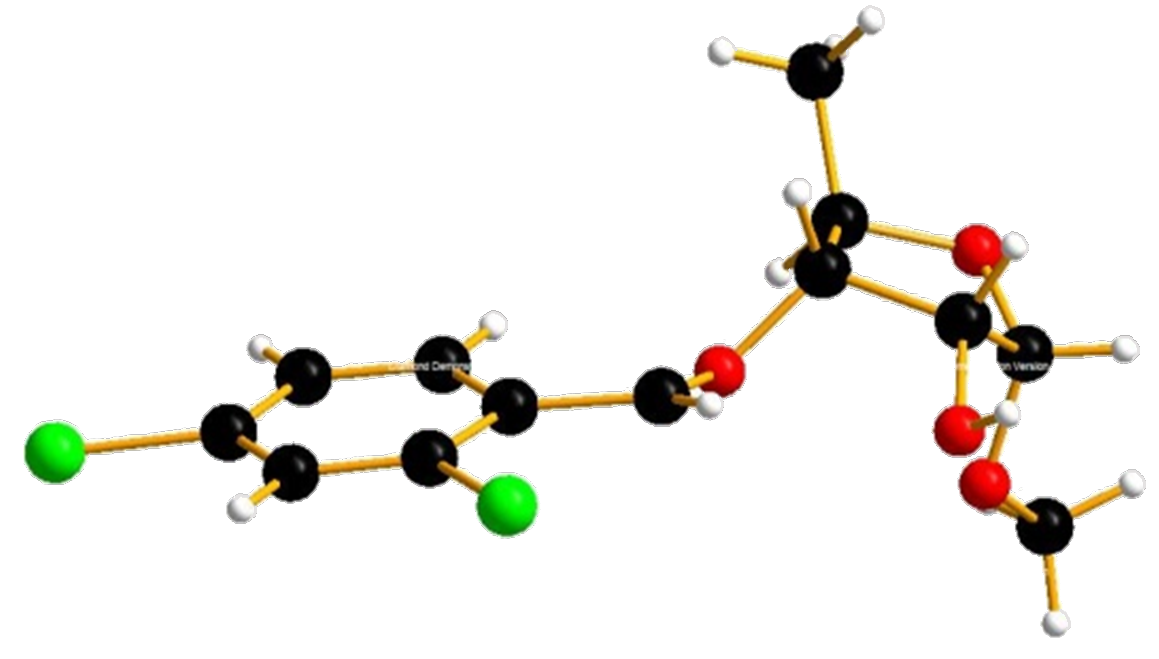
The X-ray crystal structural of 1-*O*-methyl-3-*O*-(2,4-dichlorobenzyl)-5-deoxy-α-D-ribofuranose (**9**).

Regioselective cleavage of the 2-*O*-(2,4-dichlorobenzyl) group was attempted with anhydrous SnCl_4_ in DCM by the method reported by Brown [31–32] and our group [16]. To our delight, this approach can afford 3-*O*-(2,4-dichlorobenzyl)-1-*O*-methyl-α-D-ribofuranose (**9**). After extensive optimization, compound **9** was obtained as an α-anomer only in 87% yield with exclusive regio- and stereoselectivity (Scheme 1). The anomeric hydrogen atom of **9** shows a characteristic doublet with a coupling constant of *J* = 4.8 Hz at 4.87 ppm. The structure of **9** was further unambiguously confirmed by X-ray diffraction analysis (Figure 3). This is the first report on the usage of SnCl_4_ as a regio- and stereoselective deprotection reagent in perbenzylated 5-deoxy-1-*O*-methylribofuranose. Based on these findings SnCl_4_ may prove useful for the regio- and stereoselective cleavage of the 2-*O*-benzyl group at perbenzylated 1-*O*-methylribofuranose analogues.

Dess–Martin oxidation of compound **9** provided ketone **10** in 92% yield. Next, the addition reaction with CH_3_MgBr in dry ether gave **11** exclusively with the requisite β-methyl configuration at C-2. NOE experiments showed correct correlations of H-1, H-3, 5-CH_3_ and 2-β-methyl. Then, the remaining 3-(*O*-2,4-dichlorobenzyl) group was removed under hydrogenation with 20% Pd(OH)_2_/C as a catalyst to give **12** in 91% yield. As the anomerization of the 1-*O*-α-methyl glycoside may occur under acidic conditions, the structural characterization may be much more complex. The addition of triethylamine is necessary to neutralize the possibly formed hydrogen chloride due to the reduction of the aromatic chloro substitutes during hydrogenation. The protection of all free hydroxy groups of **12** as benzoates afforded **13** in 92% yield. Due to the steric environment of 2-OH the benzoylation with benzoyl chloride required an extended reaction time (48 h) and 2 equiv DMAP as base and catalyst. At last, the 1-*O*-methyl group was transformed to 1-*O*-acetate glycosylation acceptor **4** as an anomeric mixture (α:β = 2:3) in 84% yield. Therefore, the key intermediate **4** was synthesized from commercial available 5-deoxy-1,2,3-tri-*O*-acetyl-β-D-ribofuranose (**6**) in 8 steps in 43.2% overall yield.

**Scheme 2:**
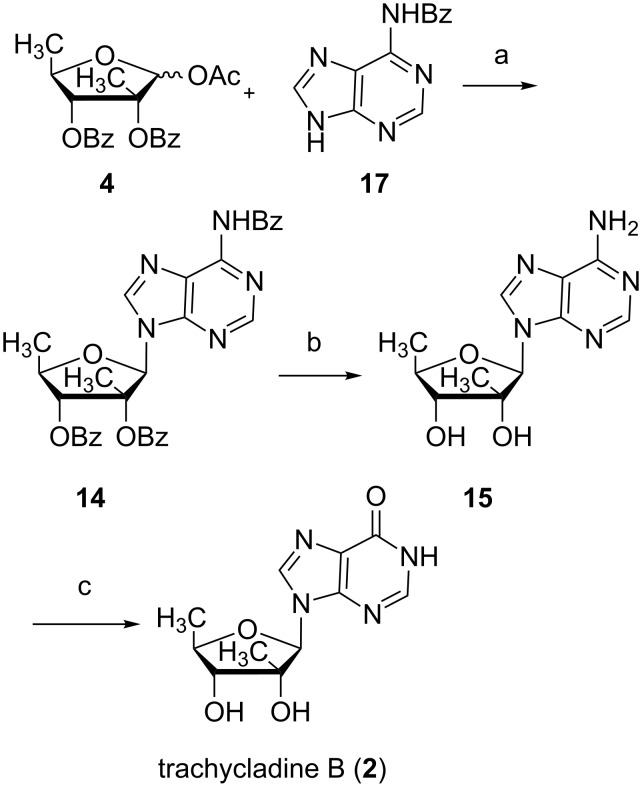
Synthesis of trachycladine B (**2**). Reagents and conditions: (a) i. *N*,*O*-Bis(trimethylsilyl)acetamide (BSA), MeCN; ii. TMSOTf, MeCN, 87%; (b) NH_3_ sat. methanol, 96%; (c) AMPDA, phosphate buffer at pH 5.6 and 40 °C, 97%.

Unfortunately, the Vorbrüggen glycosylation of carbohydrate **4** with bis(trimethylsilyl)hypoxanthine with TMSOTf as a catalyst afforded a mixture of inseparable N9 and N7 isomers with 40% yield. To further improve the yield and add to the molecular diversity, *N*^6^-benzoyladenine **17** was used as a base to give nucleoside **14** in 87% yield (Scheme 2). All benzoyl protecting groups were removed simultaneously by using ammonia saturated methanol in a sealed press tube to give **15** in 96% yield. In the next transformation to trachycladine B the classic procedure with sodium nitrite in acetic acid proved to be inefficient as it gave complicated mixtures. Therefore, we applied an alternative method.

In the past decades, adenosine deaminase (adenosine aminohydrolase, ADA, EC 3.5.4.4) and adenylate deaminase (5′-adenylic acid deaminase, AMP deaminase, AMPDA, EC 3.5.4.6) have been widely used in enzymatic hydrolysis [33–36]. In our experiment, ADA failed to give trachycladine A due to its lack of the 5′-hydroxy group, which plays a crucial role for the activity of the enzyme [35]. AMPDA is commercially available as a practical lyophilate powder and was widely used in the food industry for the production of inosine 5′-phosphate. AMPDA is a more versatile biocatalyst compared to ADA, since it can convert a larger number of adenosine derivatives into the corresponding inosine derivatives.

AMPDA was used as a biocatalyst in the enzymatic hydrolysis of adenosine **15**. The reaction proceeded smoothly in the presence of AMPDA in phosphate buffer at pH 5.6 and 40 °C with 3% DMSO as a co-solvent. Adenosine **15** was enzymatically deaminated to give trachycladine B in almost quantitative yield. The reaction is a novel instance of the broad substrate tolerance of AMPDA and extends its usage in nucleoside chemistry. Therefore, trachycladine B was synthesized in 35.0% overall yield from 5-deoxy-1,2,3-tri-*O*-acetyl-β-D-ribofuranose (**6**).

**Scheme 3:**
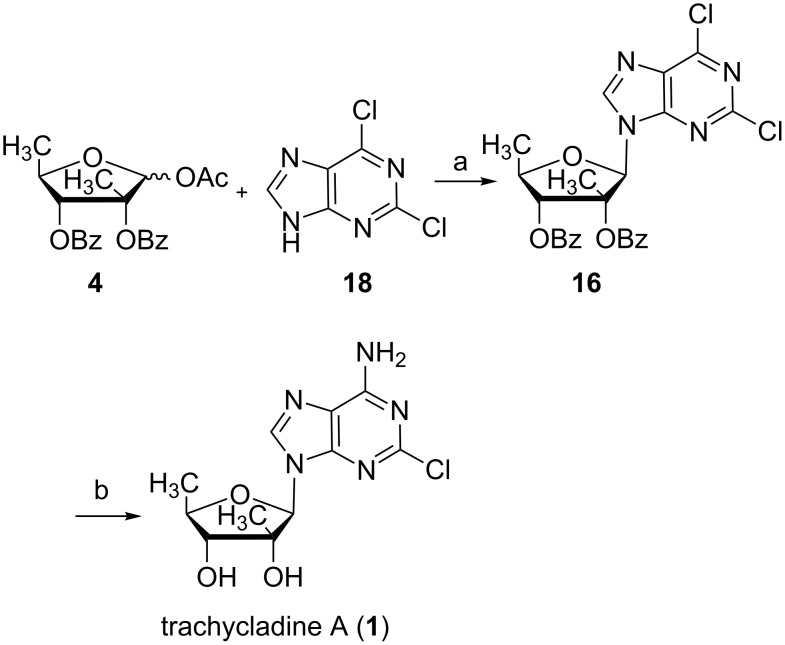
Synthesis of trachycladine A (**1**). Reagents and conditions: (a) DBU, TMSOTf, CH_3_CN, 86%; (b) NH_3_ sat. methanol, sealed press tube, 92%.

Next, the coupling of carbohydrate **4** with 2,6-dichloropurine (**18**) afforded nucleoside **16** in 86% yield with DBU as a base and TMSOTf as a Lewis acid (Scheme 3). After heated in a pressure tube with ammonia saturated methanol, the benzoyl protecting groups were removed and the chlorine atom at position C6 was substituted at the same time to afford trachycladine A in 92% yield. Therefore, trachycladine A was synthesized in 34.2% overall yield from 5-deoxy-1,2,3-tri-*O*-acetyl-β-D-ribofuranose (**6**).

## Conclusion

In conclusion, the total synthesis of trachycladine A and B have been successfully accomplished for the first time with 34.8% and 35.6% overall yield, respectively. All spectral data of synthetic trachycladine A and B are in accordance with those of the natural products. We would like to particularly emphasize three aspects of this work : (1) this is the first report using SnCl_4_ as a regio- and stereoselective deprotection reagent in perbenzylated 1-*O*-methyl-5-deoxyribofuranosides, (2) adenylate deaminase (EC 3.5.4.6) facilitated chemoenzymatic hydrolysis was used for the synthesis of trachycladine B, and (3) the reported carbohydrate **4** (1-*O*-acetyl-2,3-*O*-dibenzoyl-2-β-*C*-methyl-5-deoxy-D-ribofuranose) can be used as a common intermediate for the synthesis of versatile 5′-deoxy-2′-*C-*branched nucleosides.

## Supporting Information

File 1 Detailed experimental procedures, characterization data of compounds, and copies of ^1^H and ^13^C NMR spectra.

File 2 X-ray crystal structural of compound **9**.
