# Supplementary material for: Concise total synthesis of two marine natural nucleosides: trachycladines A and B
Source: Beilstein J Org Chem. 2014 Jul 24;10:1681–5. doi: 10.3762/bjoc.10.176 (PMC4142848; doi:10.3762/bjoc.10.176)

# Supporting Information

**for**

# Concise total synthesis of two marine natural nucleosides: trachycladines A and B

Haixin Ding[1], Wei Li[1], Zhizhong Ruan[1], Ruchun Yang[1], Zhijie Mao[1], Qiang Xiao*[1] and Jun Wu*[1,2]

Address: [1]Jiangxi Key Laboratory of Organic Chemistry, Jiangxi Science & Technology Normal University, Nanchang 330013, China and [2]Marine Drugs Research Center, College of Pharmacy,Jinan University, Guangzhou 510632, China

Email: Qiang Xiao*- xiaoqiang@tsinghua.org.cn; Jun Wu*- wwujun2003@yahoo.com

*Corresponding author

# Experimental part

**Contents:**

**General information**..........................................................................................**S2**

**Experimental and characterization section**.......................................**S2–S12**

**References**.................................................................................................**S12**

**Copies of $^{1}$H and $^{13}$C NMR spectra**..................................................**S13–S25**

**General information:**

All reagents and catalysts were purchased from commercial sources (Acros or Aldrich) and used without purification. DCM, and $CH_3CN$ were dried over $CaH_2$ and distilled prior to use. $Et_3N$ was dried over NaH and distilled prior to use. Ether was dried over $LiAlH_4$ and distilled prior to use. Thin-layer chromatography was performed using silica gel GF-254 plates with detection by UV (254 nm) or charting with 10% sulfuric acid in ethanol. Column chromatography was performed on silica gel (200–300 mesh, Qing-Dao Chemical Company, China). NMR spectra were recorded on a Bruker AV400 spectrometer, and chemical shifts (δ) are reported in ppm. $^1H$ NMR and $^{13}C$ NMR spectra were calibrated with TMS as internal standard, and coupling constants (*J*) are reported in Hz. The ESI-HRMS were obtained on a Bruker Dalton micro TOFQ II spectrometer in positive ion mode. Melting points were measured on an electrothermal apparatus and are uncorrected. Optical rotation values were measured with a Rudolphautopol IV polarimeter.

**Experimental and characterization section:**

**Synthesis of 1-*O*-methyl-2,3-*O*-di-(2,4-dichlorobenzyl)-5-deoxy-D-ribofuranose (8)**

Compound **8** was prepared in a similar manner to a previous report [1]. To a stirred solution of **7** (4.5 g, 30.4 mmol) and 2,4-dichlorobenzyl chloride (17.1 mL, 121.5 mmol) in dry DMF (50 mL) was added NaH (60% dispersion in

mineral oil, 4.86 g, 121.5 mmol) in portions at 0 °C. After addition, the mixture was allowed to warm up to rt and stirred for another 12 h. Iced water (200 mL) was added carefully to quench the reaction. The oil layer was separated and the solution was extracted with DCM (2 × 200 mL). The combined organic layer was washed with $H_2O$ (2 × 100 mL), brine (2 × 100 mL), and dried with anhydrous $Na_2SO_4$. After filtration, the filtrate was evaporated to dryness under reduced pressure. The obtained residue was purified by column chromatography (silica gel, PE–DCM, 30:1-20:1 ) to afford **8** as light yellow oil (13.6 g, 95%, mixture of α, β).

$R_f$ = 0.24 (β-anomer), $R_f$ = 0.10 (α-anomer) (PE-DCM, 30:1, v:v), β-anomer：$^1$H NMR (400 MHz, $CDCl_3$): δ 7.44~ 7.40 (m, 2H), 7.34~ 7.33 (m, 2H), 7.20 ( td, J = 8.0Hz, 2.0Hz, 2H), 4.95 (s, 1H), 4.72 (d, *J* = 13.2 Hz, 1H), 4.64(d, *J* = 12.8 Hz, 1H), 4.60 (s, 2H), 4.31~4.24 (m, 1H), 3.97 (d, *J* = 4.4 Hz, 1H), 3.87~3.85 (m, 1H), 3.38 (s, 3H, $OCH_3$), 1.37 (d, *J* = 6.4 Hz, 3H, $CH_3$); $^{13}$C NMR (100 MHz, $CDCl_3$):δ 134.2, 133.9, 133.3, 133.2, 130.0, 129.9, 129.0, 128.9, 127.1, 105.9, 84.0, 81.0, 77.2, 69.0, 68.8, 55.0, 20.7.

α-anomer：$^1$H NMR (400 MHz, $CDCl_3$):δ 7.49 (dd, *J* = 8.1 Hz, 2.0 Hz, 2H), 7.35 (dd, *J* = 8.0 Hz, 2.0 Hz, 2H), 7.22~7.19 (m, 2H), 5.00 (d, *J* = 4.4 Hz, 1H), 4.76 (dd, *J* =13.2, 2.4Hz, 2H), 4.65(dd, *J* =13.2, 4.0 Hz, 2H), 4.28~4.22 (m, 1H), 3.96~3.93 (m, 1H), 3.61~3.59 (m, 1H, H-3), 3.47 (s, 3H, $OCH_3$), 1.28 (d, *J* = 6.4 Hz, 3H, $CH_3$); $^{13}$C NMR(100 MHz, $CDCl_3$):δ 134.6, 134.2, 133.9, 133.7, 133.3, 133.2, 130.2, 129.0, 128.9, 127.2, 127.1, 102.1, 81.2, 78.6, 77.8, 69.1,

69.0, 55.4, 19.8; ESI-MS m/z: 467.3 $[M+H]^+$; HRMS: *m/z* $[M+H]^+$ calcd for $C_{20}H_{21}Cl_4O_4$: 465.0188; found: 465.0195.

**Synthesis of 1-*O*-methyl-3-*O*-(2,4-dichlorobenzyl)-5-deoxy-α-D-ribofuranose (9)**

To a solution of **8** (10.0 g, 21.3 mmol) in dry DCM (250 mL) was added 1 M $SnCl_4$ in $CH_2Cl_2$ (21.3 mL) dropwise at 0 °C under argon. After addition, the mixture was stirred for another 24 h and quenched with iced water (50 mL) carefully. Another batch DCM (200 mL) was added to dilute the above solution. The organic layer was separated and washed with $H_2O$ (2 × 100 mL), 0.2 M HCl (1 × 100 mL), sat. $NaHCO_3$ (1 × 100 mL), brine (2 × 150 mL) and dried with anhydrous$Na_2SO_4$. After filtration, the filtrate was evaporated to dryness under reduced pressure. The obtained residue was purified by column chromatography (silica gel, PE–DCM, 1:2 ) to afford **9** (5.72 g, 87%, only α-anomer) as white solid.

$R_f$ = 0.14 (PE:DCM，1:1, v:v); $[\alpha]_D^{20}$ +111.50 (c = 1.00, $CHCl_3$); $^1H$ NMR (400 MHz, $CDCl_3$):δ 7.44 (d, *J* = 8.4 Hz, 1H), 7.37 (d, *J* = 2.0 Hz, 1H), 7.27~7.25 (m, 1H), 4.87 (d, *J* = 4.8 Hz, 1H), 4.77 (d, *J* = 13.2 Hz, 1H), 4.65 (d, *J* = 13.2 Hz, 1H), 4.22~4.11 (m, 2H), 3.49~3.47 (m, 1H), 3.46 (s, 3H, $OCH_3$), 2.89 (d, *J* = 10.8 Hz, 1H), 1.27 (d, *J* = 6.4 Hz, 3H, $CH_3$); $^{13}C$ NMR (100 MHz, $CDCl_3$):δ 134.3, 134.0, 133.5, 130.0, 129.1, 127.2, 102.5, 81.6, 77.8, 71.6, 69.5, 55.3, 19.7; ESI-MS m/z: 331.0 $[M+Na]^+$; HRMS: *m/z* $[M+Na]^+$ calcd for $C_{13}H_{16}Cl_2NaO_4$: 329.0318; found: 329.0323.

**Synthesis of 1-*O*-methyl-3-*O*-(2,4-dichlorobenzyl)-2-ketone-5-deoxy-α-D-ribofuranose (10)**

To a stirred solution of **9** (5.0 g, 16.2 mmol) in dry DCM (50 mL) was added DMP (1,1,1-Triacetoxy-1,1-dihydro-1,2-benziodoxol-3(1*H*)-one) (9.7 g, 22.9mmol) portion wise. After addition, the reaction mixture was refluxed for 4 h. TLC detection showed the reaction was finished. The reaction mixture was filtered with Celite. The obtained solution was diluted with DCM (200 mL) and washed with *sat.* $NaHCO_3$ (2 × 50 mL), brine (2 × 50 mL), and dried (anhydrous $Na_2SO_4$). After filtration, the filtrated was evaporated to dryness under reduced pressure. The obtained residue was purified over a silica gel column (P.E-DCM, 1:1) to afford **10** as light yellow oil (4.56 g, 92%).

$R_f$ = 0.36 (PE-DCM, 1:1, v:v); $[\alpha]_D^{20}$ +53.50 (c = 1.00, $CHCl_3$); $^1$H NMR (400 MHz, $CDCl_3$): 7.41(d, *J* = 8.4 Hz, 1H), 7.34 (d, *J* = 1.6Hz, 1H), 7.23(dd, *J* = 8.0Hz, 1.6Hz, 1H), 5.03 (d, *J* = 12.4 Hz, 1H), 4.77~4.73(m, 2H), 4.29~4.22 (m, 1H), 3.66 (d, *J* = 8.8 Hz, 1H), 3.45 (s, 3H, $OCH_3$), 1.46 (d, *J* = 6.0 Hz, 3H); $^{13}$C NMR(100 MHz, $CDCl_3$):δ 208.2, 134.3, 133.7, 133.6, 130.4, 129.1, 127.1, 98.4, 82.7, 74.3, 69.3, 55.6, 18.7; ESI-MS m/z: 345.0 $[M+K]^+$; HRMS: *m/z* $[M+H]^+$ calcd for $C_{13}H_{15}Cl_2O_4$: 305.0342; found: 305.0351.

**Synthesis of 1-*O*-methyl-3-*O*-(2,4-dichlorobenzyl)-2-β-*C*-methyl-5-deoxy-α-D-ribofuranose (11)**

To a stirred solution of compound **10** (2.50 g, 8.14 mmol) in dry diethyl ether (20 mL) was slowly added methylmagnesium bromide (3 M solution in diethyl

ether, 6.8 mL) dropwise at −10 °C under argon. After addition, the reaction mixture was stirred overnight and quenched with *sat.*$NH_4Cl$ (20 mL). The reaction mixture was diluted with ether (150 mL).After the organic layer was separated, it was washed with $H_2O$ (2 × 50 mL), brine (2 × 50 mL) and dried with anhydrous $Na_2SO_4$. After filtration, the filtrate was evaporated under reduced pressure. The residue was purified by column chromatography (silica gel, PE-DCM, 1:2 ) to afford **11**as a light yellow oil (2.29 g，87%).

$R_f$ = 0.37 (P.E-DCM, 1:2, v:v); $[\alpha]_D^{20}$ +89.5 (c =1.00, $CHCl_3$); $^1H$ NMR (400 MHz, $CDCl_3$):δ 7.44 (d, *J* = 8.0 Hz, 1H), 7.38 (s, 1H), 7.27 (d, *J* = 7.6 Hz, 1H), 4.83 (d, *J* = 13.2 Hz, 1H), 4.63 (d, *J* = 13.2Hz, 1H), 4.48 (s, 1H), 4.12~4.10(m, 1H), 3.44 (s, 3H, $OCH_3$), 3.33 (s, 1H), 3.07 (d, *J* = 2.0 Hz, 1H), 1.36 (s, 3H,), 1.32 (d, *J* = 6.8 Hz, 3H); $^{13}C$ NMR(100 MHz, $CDCl_3$): δ 133.5, 132.9, 132.6, 129.1, 128.1, 126.1, 106.3, 86.8, 77.7, 68.7, 54.0, 24.5, 18.9; ESI-MS m/z: 359.1 $[M+K]^+$; HRMS: *m/z* $[M+H]^+$ calcd for $C_{14}H_{19}Cl_2O_4$: 321.0655; found: 321.0661.

**Synthesis of 1-*O*-methyl-2-β-*C*-methyl-5-deoxy-α-D-ribofuranose (12)**

To a solution of **11** (1.50 g, 4.64 mmol) in EtOAc (10 mL) and THF (10 mL) was added 20% $Pd(OH)_2$/C (326 mg, 0.46 mmol) and $Et_3N$ (1.36 mL, 9.75 mmol). The mixture was hydrogenated under a $H_2$ balloon at rt. After 5 h, TLC detection showed the reaction was finished. The mixture was filtered with Celite. The filtrate was concentrated to dryness under reduced pressure. The

obtained residue was purified by flash column chromatography to afford **12** as colourless oil (687 mg, 91%).

$R_f$ = 0.25 (DCM-MeOH, 200:1, v:v); $[\alpha]_D^{20}$ +94.4 (c = 0.27, $CHCl_3$); $^1$H NMR (400 MHz, $CDCl_3$): δ 4.58(s, 1H), 3.90~3.84 (m, 1H), 3.45~3.43 (m, 4H), 3.19 (t, *J* = 6.4 Hz, 1H), 2.76 (d, *J* = 7.2Hz, 1H), 1.33~1.31 (m, 6H); $^{13}$C NMR(100 MHz, $CDCl_3$): δ 106.6, 80.6, 79.5, 75.9, 55.3, 34.4, 18.5; ESI-MS m/z: 185.2 $[M+Na]^+$; HRMS: *m/z* $[M+H]^+$ calcd for $C_7H_{15}O_4$:163.0965; found:163.0971.

**Synthesis of 1-*O*-methyl-2,3-*O*-dibenzoyl-2-β-*C*-methyl-5-deoxy-α-D-ribofuranose (13)**

To a stirred solution of **12** (0.50 g, 3.08 mmol) and DMAP (0.75 g, 6.17 mmol) in dry DCM (10 mL) was added benzoyl chloride (1.43 mL, 12.3 mmol) at 0 °C. The reaction mixture was allowed to warm to room temperature and stirred for another 30 h. After the reaction was quenched with methanol (5 mL), it was evaporated in vacuo. The obtained residue was dissolved in DCM (100 mL), washed with $H_2O$ (2 × 50 mL), brine (2 ×50 mL), and dried over anhydrous $MgSO_4$. After filtration, the filtrate was eveperated to dryness under reduced pressure. The residue was purified by flash chromatography (sillica gel, P.E.-EtOAc, 20:1) afforded **13** as paled yellow oil(1.05 g, 92%).

$R_f$ = 0.34 (PE–EtOAc, 15:1, v:v ); $[\alpha]_D^{20}$ +41.3 (c = 1.00, $CHCl_3$); $^1$H NMR (400 MHz, $CDCl_3$):δ 8.13 (d, *J* = 7.6 Hz, 2H), 7.77 (d, *J* = 8.0 Hz, 2H), 7.58 (t, *J* = 7.6 Hz, 1H), 7.46~7.39 (m, 3H), 7.16(t, *J* = 7.6 Hz, 2H), 5.26 (s, 1H), 5.01 (d, *J* = 4.0 Hz, 1H), 4.40~4.34 (m, 1H), 3.40 (s, 3H, $OCH_3$), 1.78 (s, 3H), 1.51 (d, *J*

= 6.4 Hz, 3H); $^{13}$C NMR(100 MHz, $CDCl_3$):δ 166.3, 164.8, 133.2, 132.7, 130.0, 129.7, 128.4, 128.1, 106.5, 81.9, 81.0, 76.8, 55.3, 22.8, 19.5; ESI-MS m/z: 393.2 $[M+Na]^+$; HRMS: *m/z* $[M+H]^+$ calcd for $C_{21}H_{23}O_6$: 371.1489; found: 371.1493.

**Synthesis of 1-*O*-acetyl-2,3-*O*-dibenzoyl-2-β-*C*-methyl-5-deoxy-β-D-ribofuranose (4)**

A solution of **13** (400 mg, 1.08 mmol) in acetic anhydride (5mL) and acetic acid (5 mL) was treated with 0.2 mL of concentrated sulfuric acid at 10–15 °C. After 24 h, the reaction mixture was poured into iced water (50 mL). The solution was extracted with DCM (2 × 50 mL). The combined organic layers were washed with *sat.* $NaHCO_3$ (2 × 30 mL), brine (2 × 30 mL),and dried with anhydrous $Na_2SO_4$. After filtration, the filtrate was evaporated under reduced pressure. The residue was purified over a silica gel column (PE-EtOAc, 20:1-10:1) to afford **4** as syrup (364 mg, β:α = 4:1, 84%).

$R_f$ = 0.18 (PE-EtOAc, 15:1, v:v); β-anomer: $^1$H NMR (400 MHz, $CDCl_3$):δ 8.07(t, *J* = 8.4Hz, 1H), 8.00 (d, *J* =7.6Hz, 2H), 7.80 (d, *J* = 7.6 Hz, 2H), 7.54 (t, *J* = 7.6Hz, 1H), 7.50~7.43 (m, 3H), 7.36 (t, *J* = 7.6Hz, 2H), 7.26 (t, *J* = 7.6Hz, 2H), 6.56 (s, 1H), 5.19 (s, 1H), 4.47~4.46 (m, 1H), 2.04 (s, 3H, OAc), 1.83 (s, 3H,), 1.55 (d, *J* = 6.4 Hz, 3H); $^{13}$C NMR(100 MHz, $CDCl_3$):δ 169.6, 165.8, 164.6, 133.3, 133.1, 129.7, 129.6, 128.4, 128.3, 98.8, 81.7, 80.6, 79.9, 22.2, 21.1, 19.4; ESI-MS m/z: 421.1 $[M+Na]^+$, HRMS: *m/z* $[M+Na]^+$ calcd for$C_{22}H_{22}NaO_7$:421.1258; found:421.1261.

**Synthesis of *N*[6]-benzoyl-9-(2',3'-di-*O*-benzoyl-2'-β-*C*-methyl-5'-deoxy-β-D-ribofuranosyl)adenine (14)**

To a suspension of $N^6$-benzoyladenine (204 mg, 0.853 mmol) in dry MeCN (10 mL) was added BSA (408 mg, 2.01 mmol). The mixture was heated at 60 °C for 30 min. After cooled to rt, a solution of **4** (200 mg, 0.502 mmol) in dry MeCN (3 mL) was added. TMSOTf (558 mg, 2.51 mmol) was added to the above reaction mixture at 0 $^o$C. The solution was stirred for 20 min before heating to 80 °C for 2 h. Then the reaction mixture was poured into cold sat. $NaHCO_3$ (30 mL).It was extracted with DCM (2 × 100 mL). The combined organic layers were washed with sat. aq $NaHCO_3$ (1 × 100 mL), brine (2 × 100 mL), and dried with anhydrous $Na_2SO_4$. After filtration, the filtrate was evaporated under reduced pressure. The residue was purified over a silica gel column (DCM–EtOAc, 10:1) to give nucleoside **14** as a white solid (257 mg, 87%).

$R_f$ = 0.70 (DCM-EtOAc, 5:1, v:v); $[\alpha]_D^{20}$ -135.0 (c = 0.20, $CHCl_3$);$^1$H NMR (400 MHz, $CDCl_3$): δ 9.30 (brs, 1H), 8.98 (s, 1H), 8.29 (s, 1H), 8.13 (d, J = 8.0 Hz, 2H), 8.05 (d, *J* = 8.0 Hz, 2H), 7.96 (d, *J* = 8.0 Hz, 2H), 7.60 ~7.57 (m, 2H), 7.54~7.50 (m, 3H), 7.44 (t, *J* = 8.0 Hz, 2H), 7.33 (t, *J* = 8.0 Hz, 2H), 6.76 (s, 1H), 5.80 (d, *J* = 4.8 Hz, 1H), 4.50 ~ 4.45 (m, 1H), 1.70 (d, *J* = 6.4 Hz, 3H), 1.58 (s, 3H);$^{13}$C NMR (100 MHz, $CDCl_3$): δ 165.4, 165.2, 164.6, 153.0, 151.6, 149.8, 141.6, 133.7, 133.6, 132.8, 129.9, 129.7, 129.6, 129.0, 128.9, 128.5, 128.4, 127.9, 123.4, 88.5, 84.5, 79.8, 79.2, 18.6, 18.1; ESI-MS m/z: 578.2 $[M+H]^+$, HRMS: *m/z* $[M+H]^+$ calcd for $C_{32}H_{28}N_5O_6$: 578.2034; found: 578.2036.

**Synthesis of 9-(2'-β-*C*-methyl-5'-deoxy-β-D-ribofuranosyl)adenine (15)**

A solution of **14** (180 mg, 0.312 mmol) in freshly prepared sat. methanolic ammonia (20 mL) was stirred at rt in a sealed press tube for 24 h. The reaction mixture was evaporated in vacuo to dryness. The residue was purified by flash chromatography (silica gel, DCM-MeOH, 10:1-6:1) to give nucleoside **14** as a white solid (79 mg, 96%).

$R_f$ = 0.42 (DCM-MeOH, 5:1, v:v); $[\alpha]_D^{23}$ -38.7 (c = 0.17, MeOH); $^1$H NMR (400 MHz, DMSO-$d_6$): δ 8.15(s, 1H), 8.13(s, 1H), 7.28 (s, 2H, $NH_2$), 5.89 (s, 1H), 5.20 (d, *J* = 6.4 Hz, 1H), 5.11 (s, 1H), 3.92~3.86 (m, 2H), 1.36 (d, *J* = 6.0 Hz, 3H), 0.77 (s, 3H); $^{13}$C NMR (100MHz, DMSO-$d_6$):δ 156.5, 153.0, 149.5, 139.6, 119.4, 91.5, 78.9, 78.8, 77.7, 20.5, 18.0; ESI-MS m/z: 266.1 $[M+H]^+$; HRMS: *m/z* $[M+H]^+$ calcd for $C_{11}H_{16}N_5O_3$: 266.1248; found: 266.1250.

**Synthesis of trachycladine B (2)**

Compound **15** (20 mg, 75 μmol) in phosphate buffer (5.8 mL of 0.2 mol/L $Na_2HPO_4$, 4.2 mL of 0.1 mol/L citric acid, pH 5.6) containing 3% DMSO were treated with AMP deaminase (EC 3.5.4.6，20 mg) for 1 h at 40 °C. The solution was lyophilized and the residue was purified by flash chromatography (silica gel, DCM-MeOH, 6:1) to give trachycladine B as a colorless solid (19 mg, 97%).

$R_f$ = 0.33 (DCM–MeOH, 5:1, v:v); $[\alpha]_D^{23}$ -54.9(c = 0.10, MeOH); $^1$H NMR (400 MHz, DMSO-$d_6$):δ 12.40 (brs, 1H), 8.12 (s, 1H, H-8), 8.07 (s, 1H, H-2), 5.88 (s, 1H, H-1'), 5.27 (d, *J* = 6.4 Hz, 1H, OH), 5.16 (s, 1H, OH), 3.96 ~3.92

(m, 1H, H-3’), 3.76 ~3.73 (m, 1H, H-4’), 1.37 (d, *J* = 6.0 Hz, 3H, H-5’), 0.78 (s, 3H, H-2’-CH3); $^{13}$C NMR(100 MHz, DMSO-*d*$_6$):δ 157.0, 148.3, 146.3, 138.9, 124.7, 91.7, 78.9, 77.9, 20.5,17.9;ESI-MS m/z: 267.0 [M+H]$^-$, HRMS: *m/z* [M+H]$^+$ calcd for $C_{11}H_{15}N_4O_4$: 267.1088; found: 267.1091.

**Synthesis of 2,6-dichloro-9-(2',3'-di-*O*-benzoyl-2'-β-*C*-methyl-5'-deoxy-β-D-ribofuranosyl)purine (16)**

To a stirred solution of 2,6-dichloropurine (47 mg, 0.25 mmol) and **4** (50.0 mg, 0.13 mmol) in dry MeCN (5 mL) was added DBU (74 μL, 0.50 mmol) and TMSOTf (136 μL, 0.75 mmol) in dry MeCN (2.0 mL) at 0 °C. After addition, the reaction mixture was allowed to warm to rt and stirred for another 4 h. Then it was quenched with sat. $NaHCO_3$ (30 mL). The aqueous phase was extracted with DCM (2 × 50 mL). The combined organic layers were washed with $H_2O$ (2 × 50 mL), brine (2 × 50 mL), and dried over anhydrous $MgSO_4$. After filtration, the filtrate was evaporated under reduced pressure. The residue was purified by flash chromatography (silica gel, DCM-EtOAc, 20:1) to afford nucleoside **16** as white solid (57 mg, 86%).

$R_f$ = 0.62 (DCM–EtOAc, 10:1, v:v); $[\alpha]_D^{20}$ -156.8 (c = 0.31, $CHCl_3$);$^1$H NMR (400 MHz, $CDCl_3$): 8.41 (s, 1H), 8.18 (d, *J* = 7.2 Hz, 2H), 7.87 (d, *J* = 7.6 Hz, 2H), 7.61 (t, *J* = 7.2 Hz, 1H), 7.51~7.46 (m, 3H), 7.29~7.25 (m, 2H), 6.67 (s, 1H), 5.61 (d, *J* = 3.6 Hz, 1H), 4.48~4.45 (m, 1H), 1.74 (d, *J* = 6.4 Hz, 3H), 1.55 (s, 3H); $^{13}$C NMR (100 MHz, $CDCl_3$):δ 165.2, 164.9, 153.4, 152.4, 152.3, 143.9, 133.6, 133.5, 131.3, 130.1, 129.7, 129.2, 128.8, 128.5, 128.3, 88.9, 83.4, 80.6,

79.5, 18.9, 18.0; ESI-MS m/z: 528.6 $[M+H]^-$, HRMS: *m/z* $[M+H]^+$ calcd for $C_{25}H_{21}Cl_2N_4O_5$: 527.0884; found: 527.0891.

**Synthesis of Trachycladine A (1)**

A solution of **16** (40 mg, 0.057 mmol) in methanolic ammonia (MeOH saturated with gaseous $NH_3$ at 0 °C, 10 mL) was stirred at 120 °C in an autoclave for 12 h. After cooling, the mixture was evaporated in vacuo. The residue was purified by flash chromatography (silica gel, DCM-MeOH, 10:1) to give trachycladine A as a pale yellow solid (21 mg, 92%).

$R_f$ = 0.37 (DCM-MeOH, 8:1, v:v); $[\alpha]_D^{24}$ -25.2 (c = 0.40, acetone); $^1$H NMR (400 MHz, DMSO-$d_6$): 8.20 (s, 1H), 7.84 (brs, 2H, NH2), 5.81 (s, 1H), 5.24 (d, *J* = 6.8 Hz, 1H), 5.17 (s, 1H), 3.95~3.90 (m, 1H), 3.83 (t, *J* = 8.0 Hz, 1H ), 1.37 (d, *J* = 6.0 Hz, 3H), 0.80 (s, 3H); $^{13}$C NMR (100 MHz, DMSO-$d_6$): 157.3, 153.5, 150.4, 140.3, 118.5, 91.8,78.9, 78.8, 78.0, 20.6, 17.9; ESI-MS m/z: 301.2 $[M+H]^-$, HRMS: *m/z* $[M+H]^+$ calcd for $C_{11}H_{15}ClN_5O_3$: 300.0858; found: 300.0861.

**References:**

[1] Song, Y.; Yang, R.; Ding, H.; Sun, Q.; Xiao, Q.; Ju, Y. *Synthesis* **2011,** 1213-1218.

## Copies of $^1$H and $^{13}$C NMR spectra

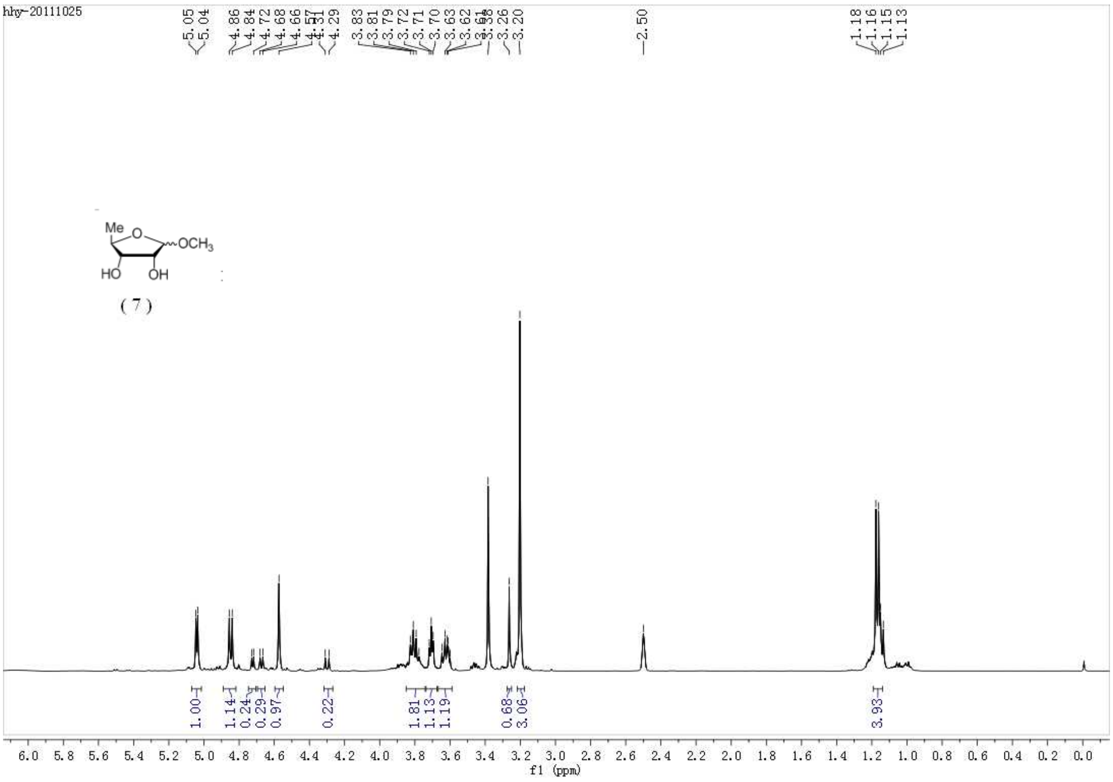


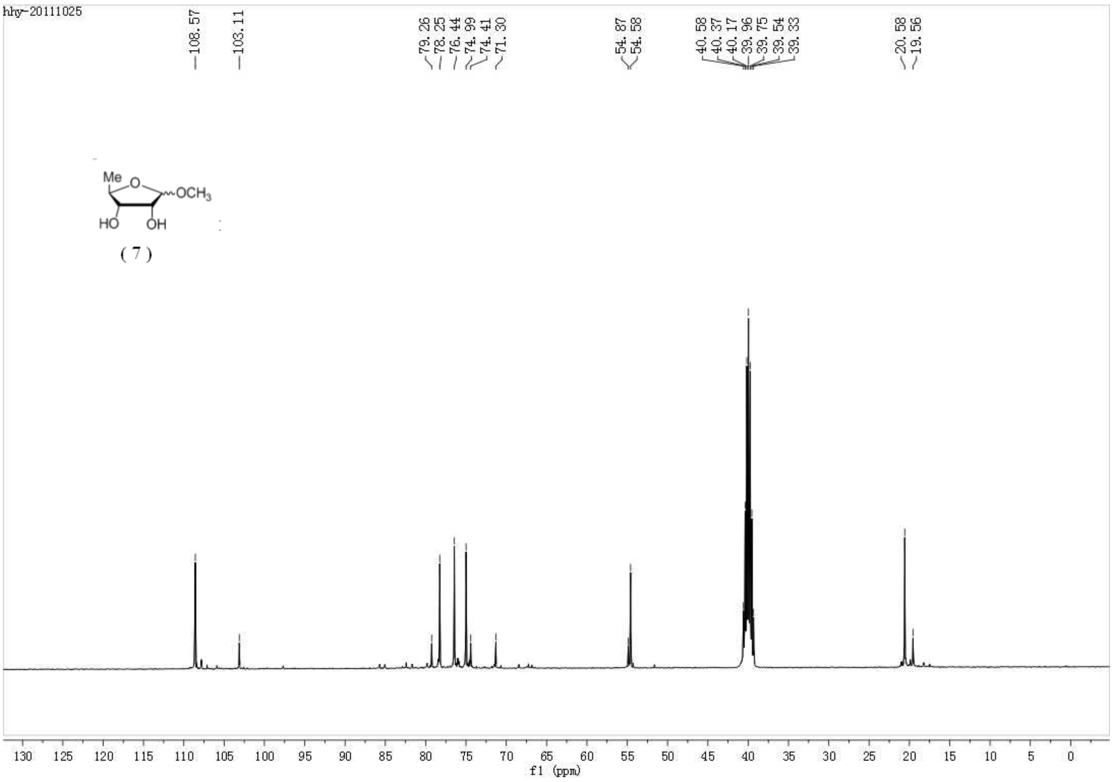

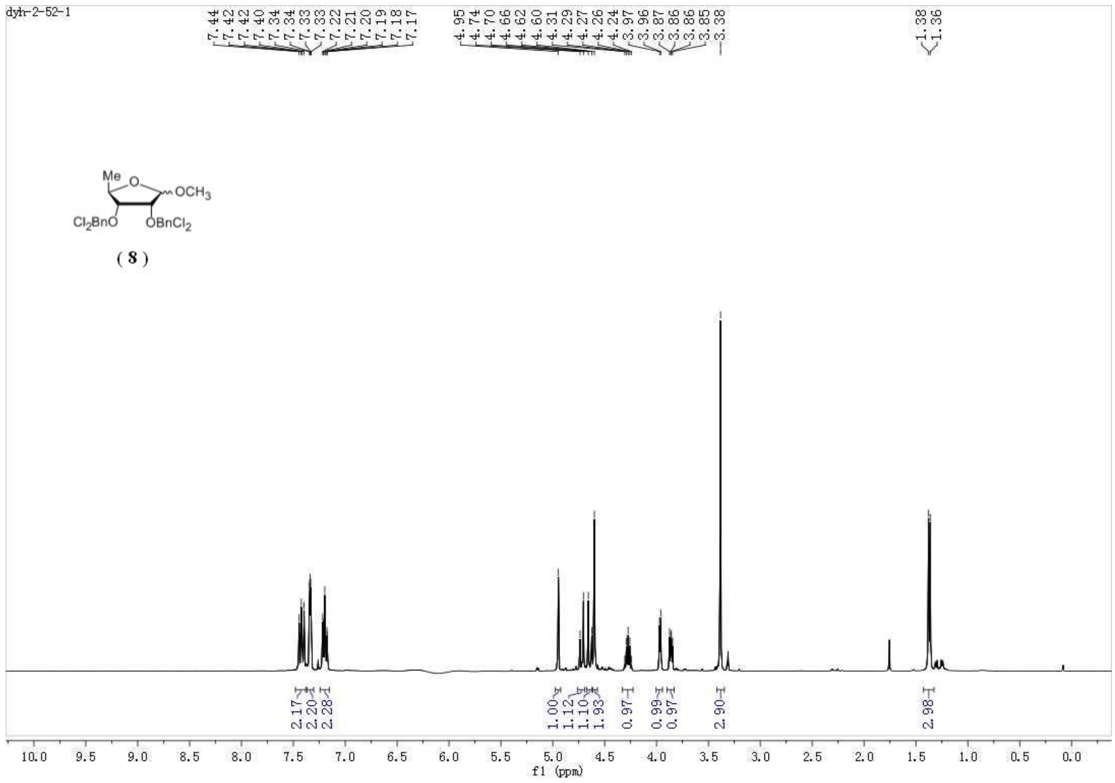
dyh-2-52-1
7.44 7.42 7.42 7.40 7.34 7.33 7.33 7.22 7.21 7.20 7.19 7.18 7.17
4.95 4.74 4.70 4.66 4.62 4.60 4.31 4.29 4.27 4.26 4.24 3.97 3.96 3.87 3.86 3.86 3.85 3.38
1.38 1.36
Me
O
OCH3
Cl2BnO
OBnCl2
( 8 )
2.17 2.20 2.28
1.00 1.12 1.10 1.93 0.97 0.99 0.97 2.90
2.98
10.0 9.5 9.0 8.5 8.0 7.5 7.0 6.5 6.0 5.5 5.0 4.5 4.0 3.5 3.0 2.5 2.0 1.5 1.0 0.5 0.0
f1 (ppm)

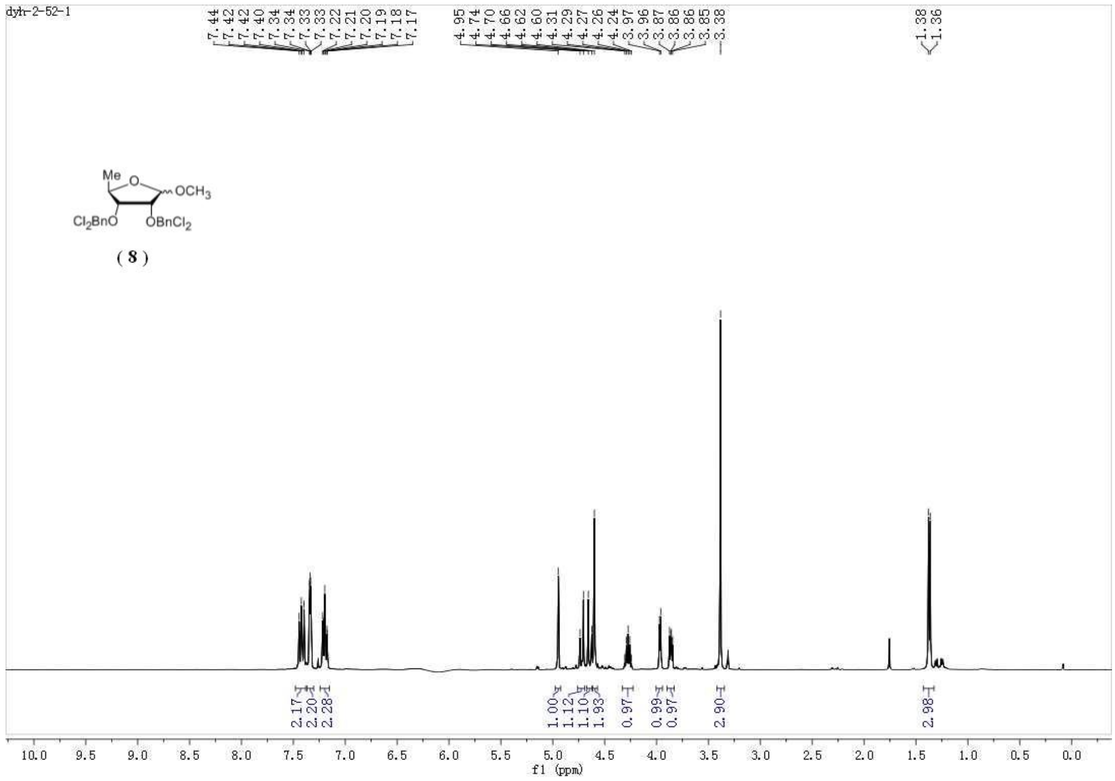
dyh-2-52-1
7.44 7.42 7.42 7.40 7.34 7.33 7.33 7.22 7.21 7.20 7.19 7.18 7.17
4.95 4.74 4.70 4.66 4.62 4.60 4.31 4.29 4.27 4.26 4.24 3.97 3.96 3.87 3.86 3.86 3.85 3.38
1.38 1.36
Me
O
OCH3
Cl2BnO
OBnCl2
( 8 )
2.17 2.20 2.28
1.00 1.12 1.10 1.93 0.97 0.99 0.97 2.90
2.98
10.0 9.5 9.0 8.5 8.0 7.5 7.0 6.5 6.0 5.5 5.0 4.5 4.0 3.5 3.0 2.5 2.0 1.5 1.0 0.5 0.0
f1 (ppm)

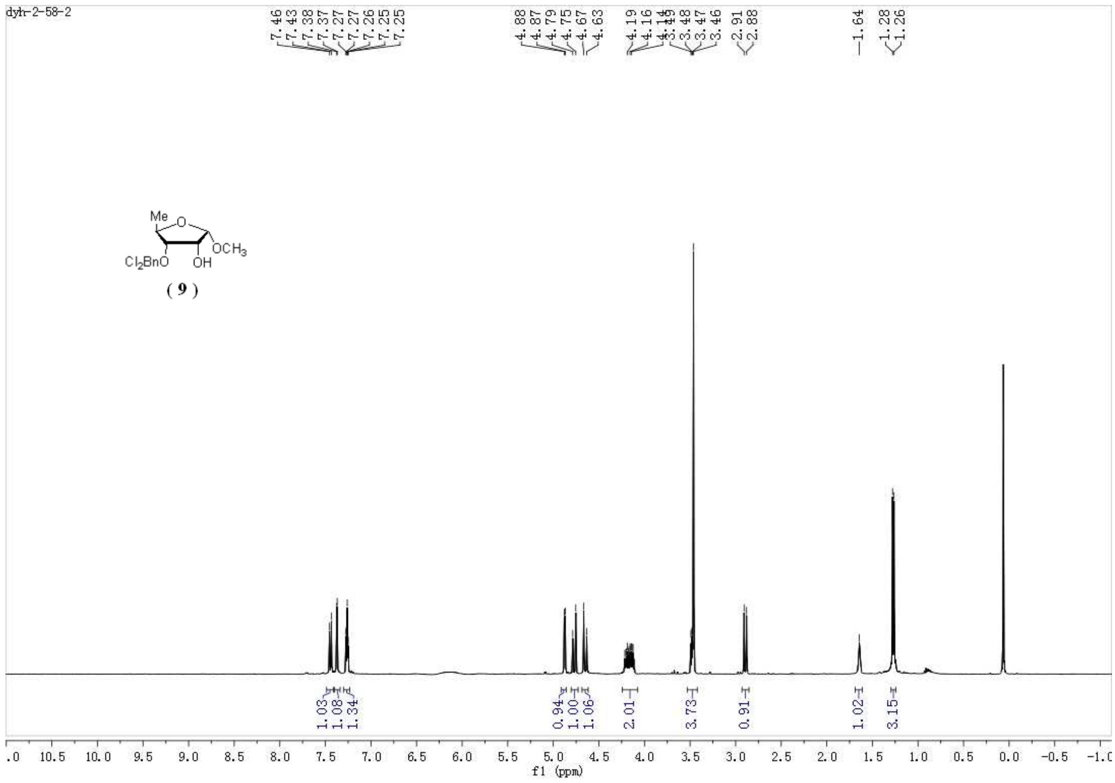
dyh-2-58-2
7.46 7.43 7.38 7.37 7.27 7.27 7.26 7.25 7.25
4.88 4.87 4.79 4.75 4.67 4.63
4.19 4.16 4.14 3.48 3.47 3.46
2.91 2.88
1.64
1.28 1.26
Me
O
OCH3
Cl2BnO
OH
( 9 )
1.03 1.08 1.34
0.94 1.00 1.06
2.01
3.73
0.91
1.02
3.15
f1 (ppm)

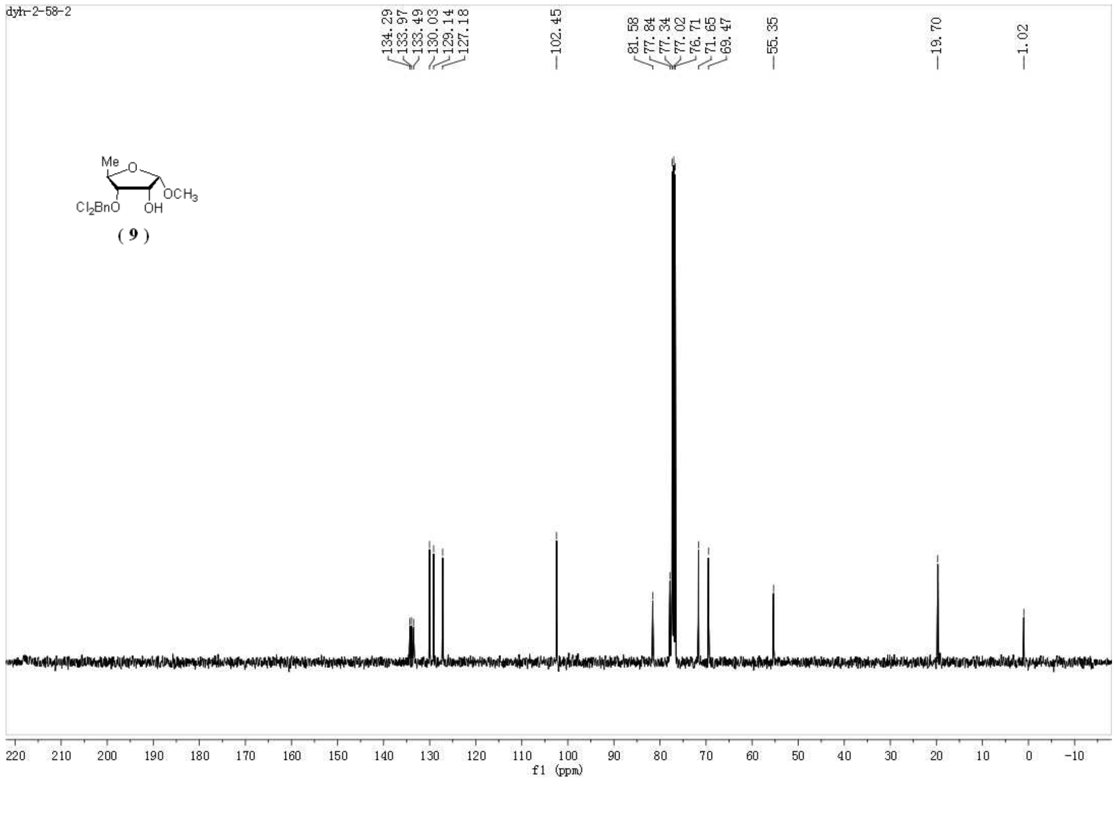
dyh-2-58-2
134.29 133.97 133.49 130.03 129.14 127.18
102.45
81.58 77.84 77.34 77.02 76.71 71.65 69.47
55.35
19.70
1.02
Me
O
OCH3
Cl2BnO
OH
( 9 )
f1 (ppm)

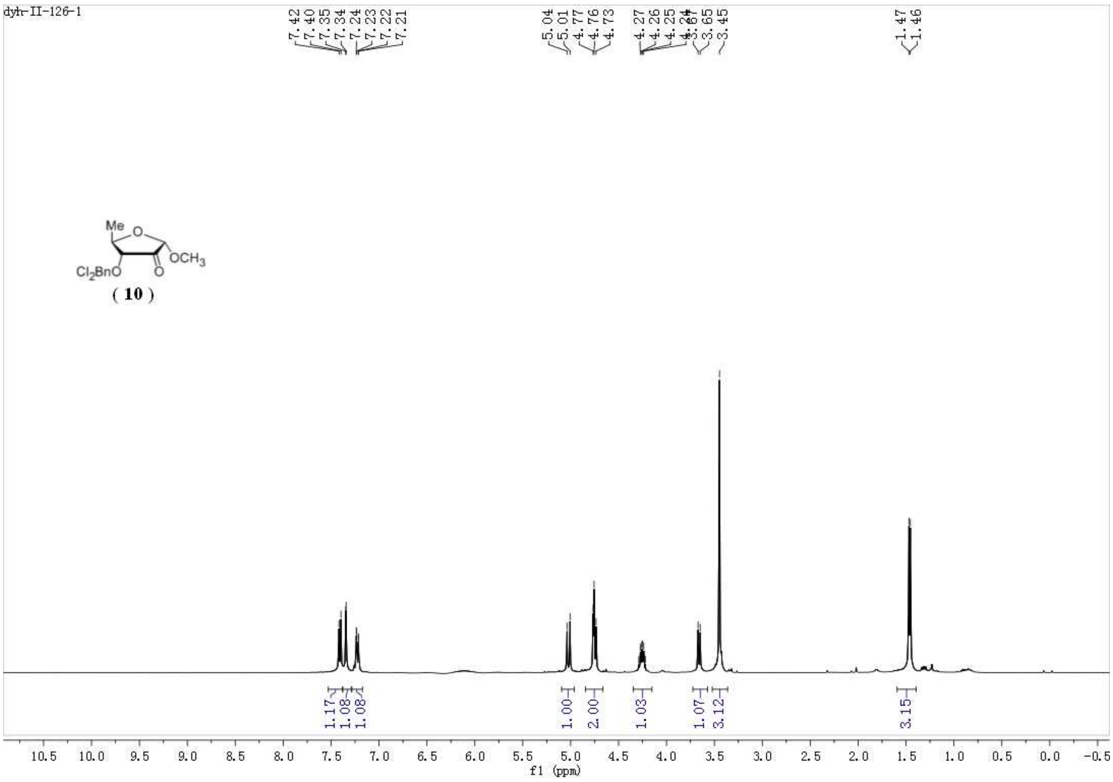
dyh-II-126-1
7.42 7.40 7.35 7.34 7.24 7.23 7.22 7.21
5.04 5.01 4.77 4.76 4.73
4.27 4.26 4.25 4.24 3.67 3.65 3.45
1.47 1.46
Me
O
OCH3
Cl2BnO
(10)
1.17 1.08 1.08 1.00 2.00 1.03 1.07 3.12 3.15
f1 (ppm)

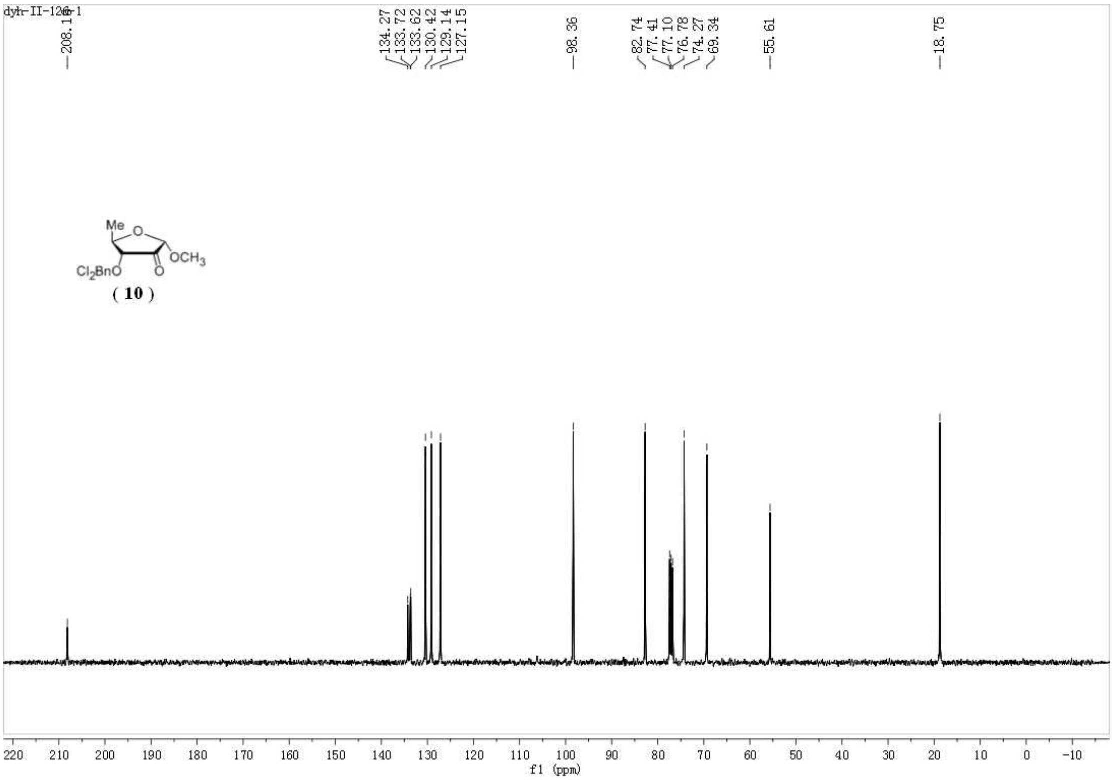
dyh-II-126-1
208.16
134.27 133.72 133.62 130.42 129.14 127.15
98.36
82.74 77.41 77.10 76.78 74.27 69.34
55.61
18.75
Me
O
OCH3
Cl2BnO
(10)
f1 (ppm)

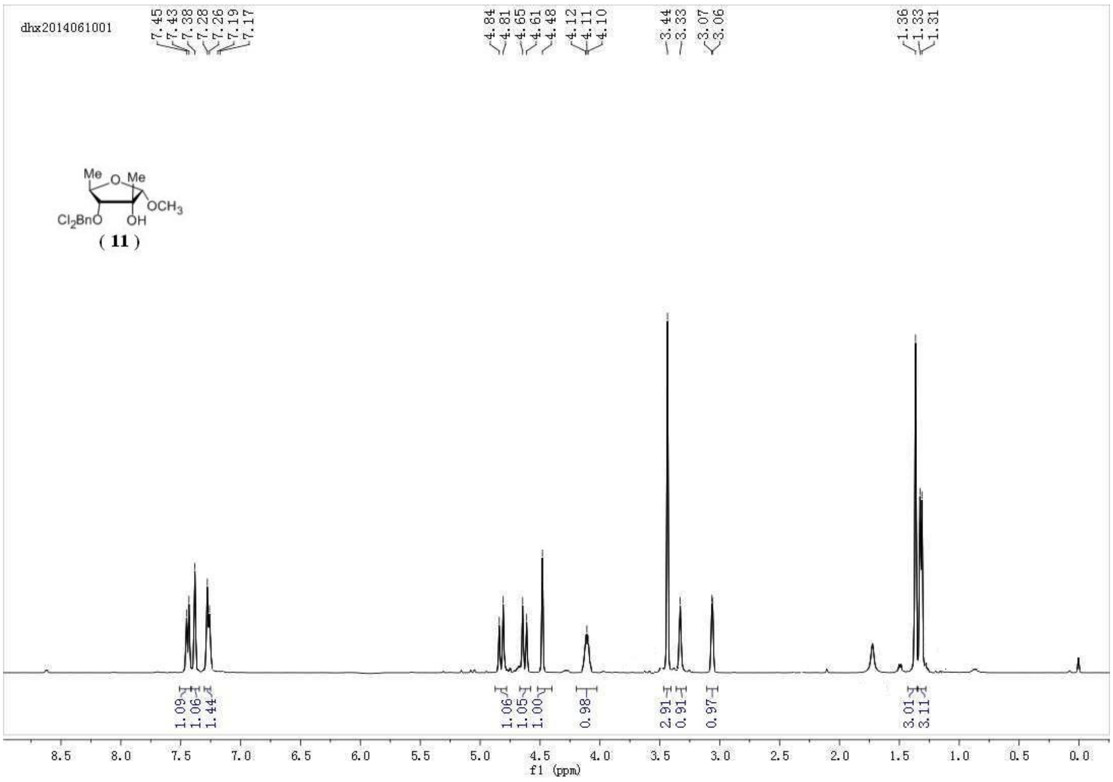
dhx2014061001
7.45
7.43
7.38
7.28
7.26
7.19
7.17
4.84
4.81
4.65
4.61
4.48
4.12
4.11
4.10
3.44
3.33
3.07
3.06
1.36
1.33
1.31
Me
O
Me
OCH3
Cl2BnO
OH
( 11 )
1.09
1.06
1.44
1.06
1.05
1.00
0.98
2.91
0.91
0.97
3.01
3.11
f1 (ppm)

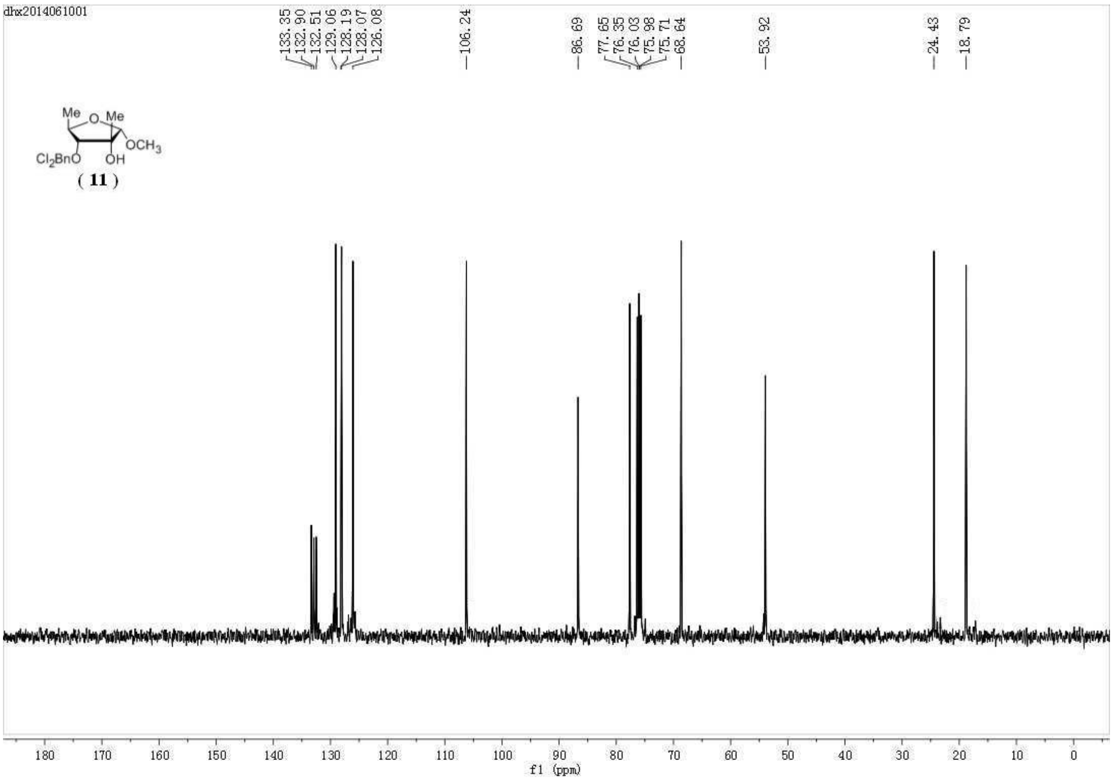
dhx2014061001
133.35
132.90
132.51
129.06
128.19
128.07
126.08
106.24
86.69
77.65
76.35
76.03
75.98
75.71
68.64
53.92
24.43
18.79
Me
O
Me
OCH3
Cl2BnO
OH
( 11 )
f1 (ppm)

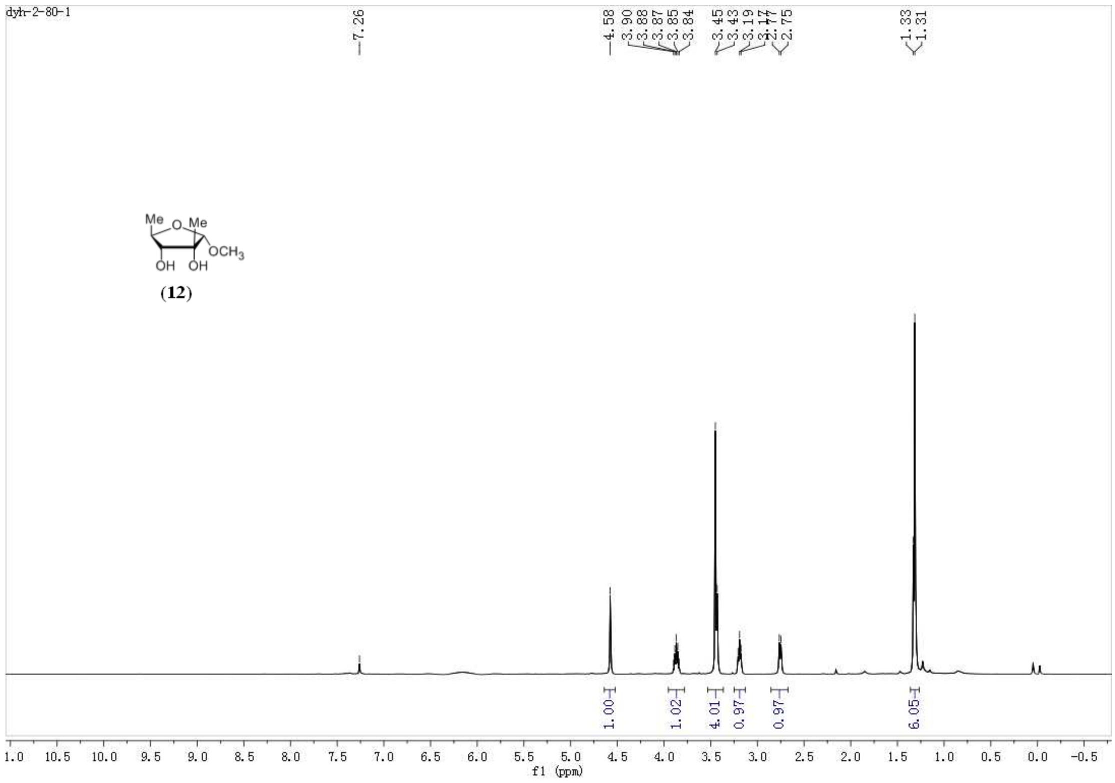
dyh-2-80-1
7.26
4.58
3.90
3.88
3.87
3.85
3.84
3.45
3.43
3.19
3.17
2.77
2.75
1.33
1.31
Me
O
Me
OCH3
OH
OH
(12)
1.00
1.02
4.01
0.97
0.97
6.05
f1 (ppm)

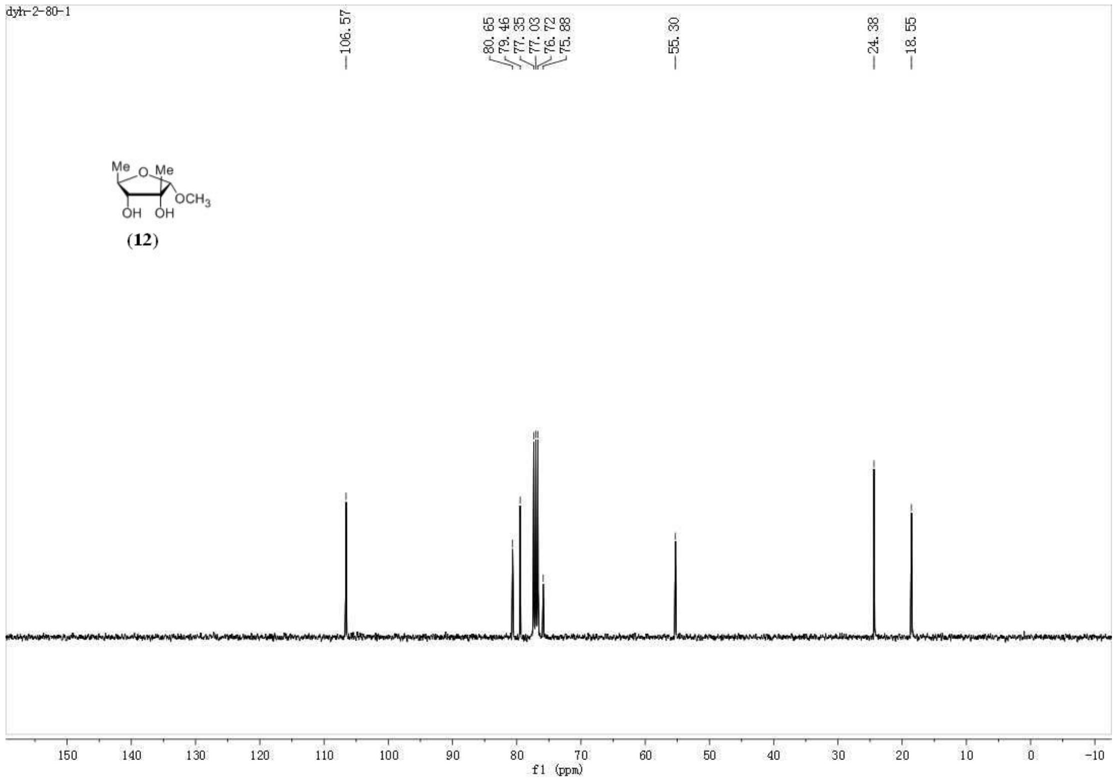
dyh-2-80-1
106.57
80.65
79.46
77.35
77.03
76.72
75.88
55.30
24.38
18.55
Me
O
Me
OCH3
OH
OH
(12)
f1 (ppm)

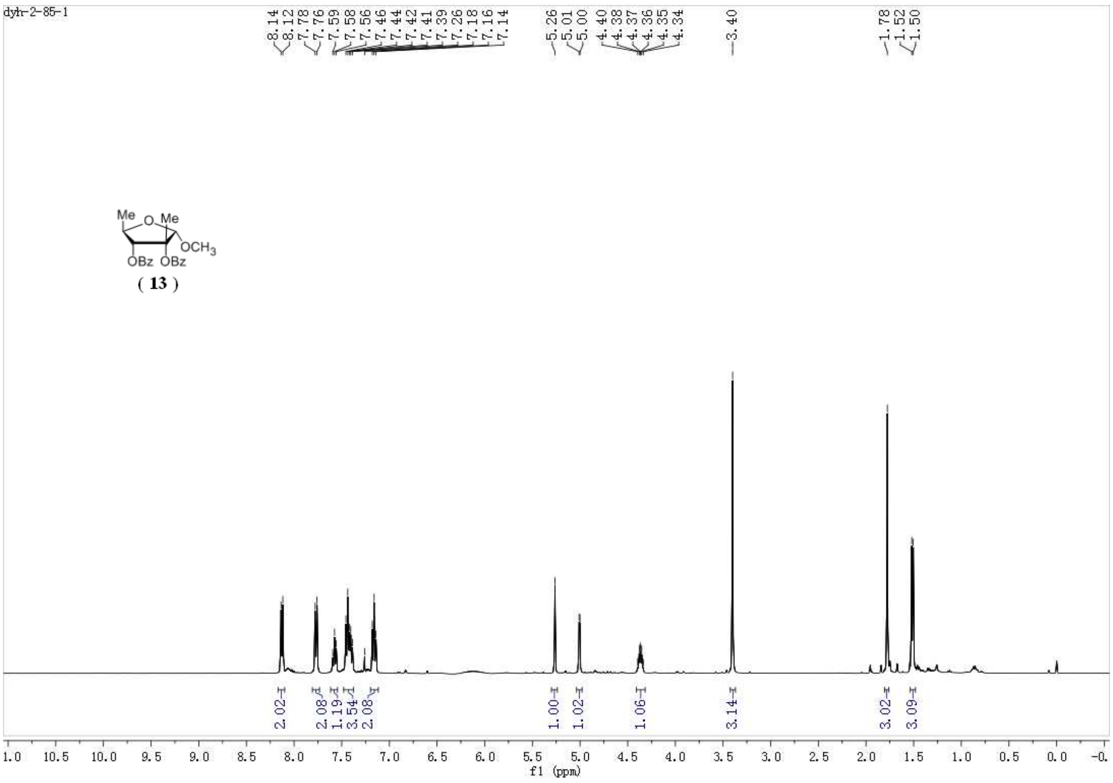
dyh-2-85-1
8.14
8.12
7.78
7.76
7.59
7.58
7.56
7.46
7.44
7.42
7.41
7.39
7.26
7.18
7.16
7.14
5.26
5.01
5.00
4.40
4.38
4.37
4.36
4.35
4.34
3.40
1.78
1.52
1.50
Me
O
Me
OCH3
OBz
OBz
( 13 )
2.02
2.08
1.19
3.54
2.08
1.00
1.02
1.06
3.14
3.02
3.09
f1 (ppm)

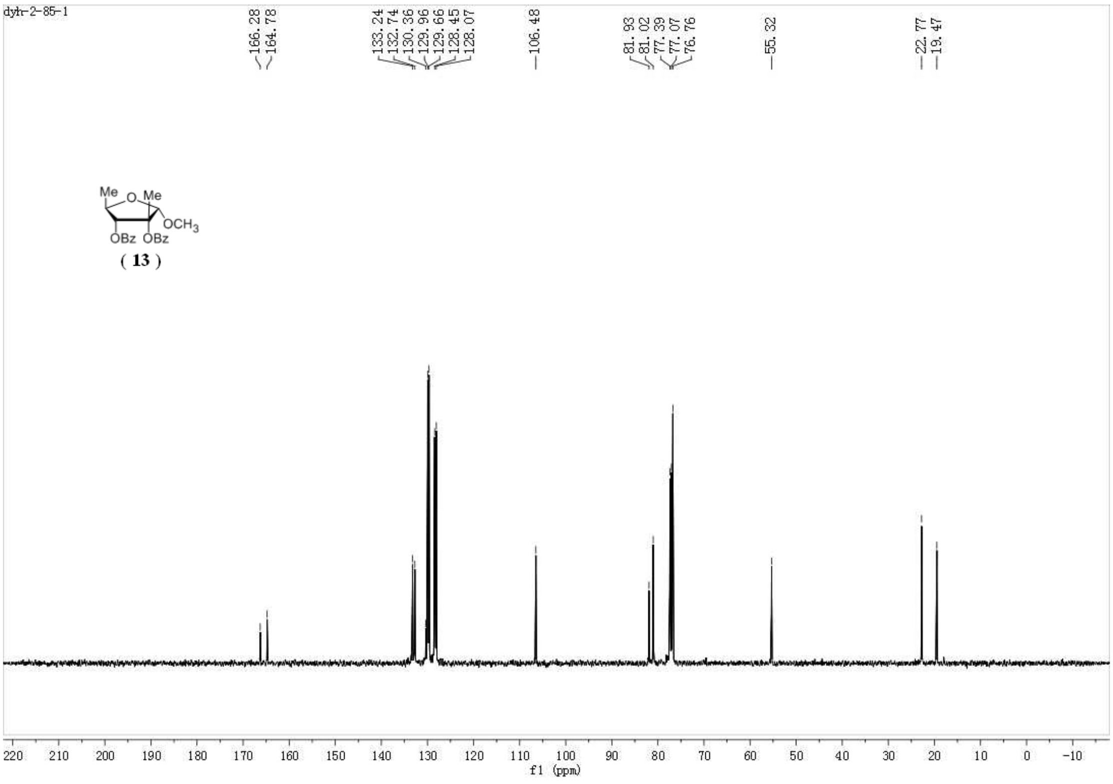
dyh-2-85-1
166.28
164.78
133.24
132.74
130.36
129.96
129.66
128.45
128.07
106.48
81.93
81.02
77.39
77.07
76.76
55.32
22.77
19.47
Me
O
Me
OCH3
OBz
OBz
( 13 )
f1 (ppm)

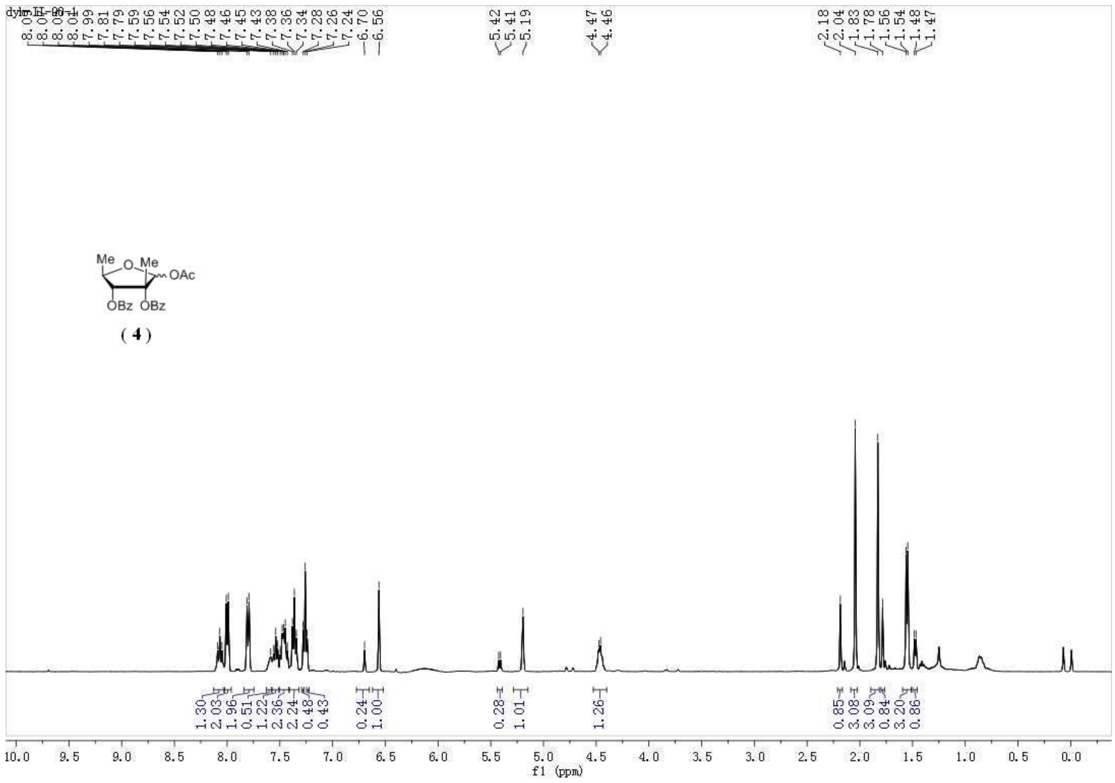
dyh-II-90-1
8.07 8.05 8.03 8.01 7.99 7.81 7.79 7.59 7.56 7.54 7.52 7.50 7.48 7.46 7.45 7.43 7.38 7.36 7.34 7.28 7.26 7.24 6.70 6.56
5.42 5.41 5.19
4.47 4.46
2.18 2.04 1.83 1.78 1.56 1.54 1.48 1.47
Me O Me OAc
OBz OBz
(4)
1.30 2.03 1.96 0.51 1.22 2.36 2.24 0.48 0.43 0.24 1.00 0.28 1.01 1.26 0.85 3.08 3.09 0.84 3.20 0.86
f1 (ppm)

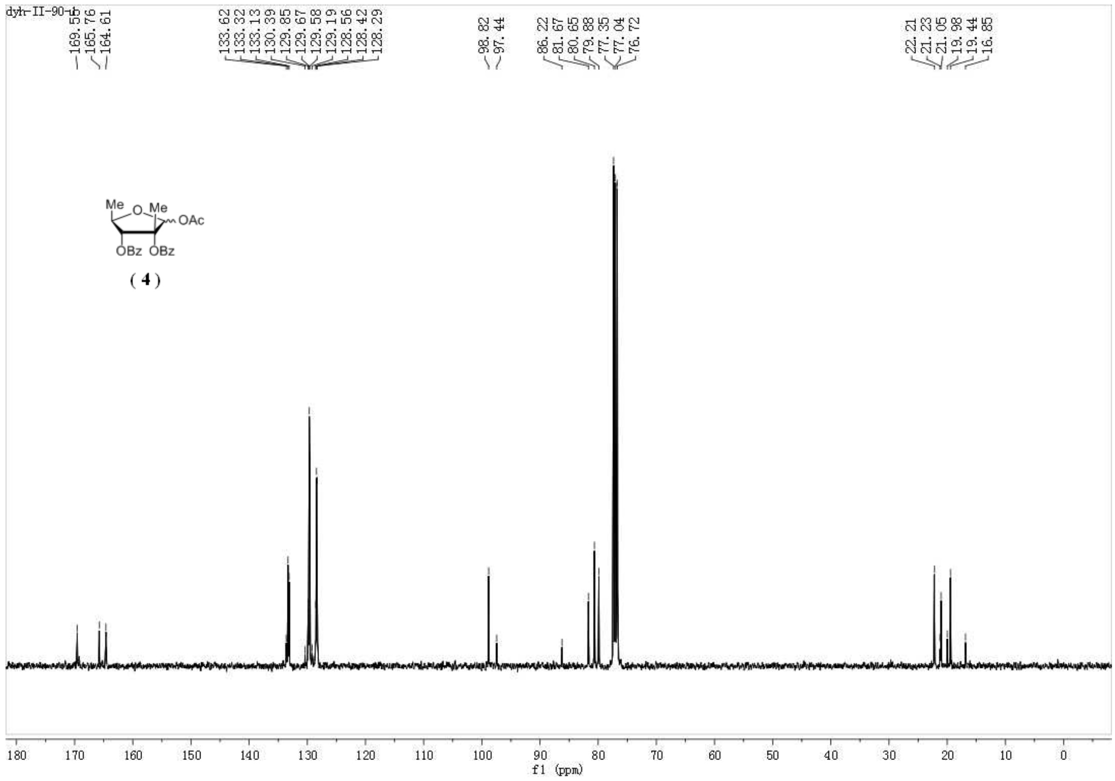
dyh-II-90-1b
169.55 165.76 164.61
133.62 133.32 133.13 130.39 129.85 129.67 129.58 129.19 128.56 128.42 128.29
98.82 97.44
86.22 81.67 80.65 79.88 77.35 77.04 76.72
22.21 21.23 21.05 19.98 19.44 16.85
Me O Me OAc
OBz OBz
(4)
f1 (ppm)

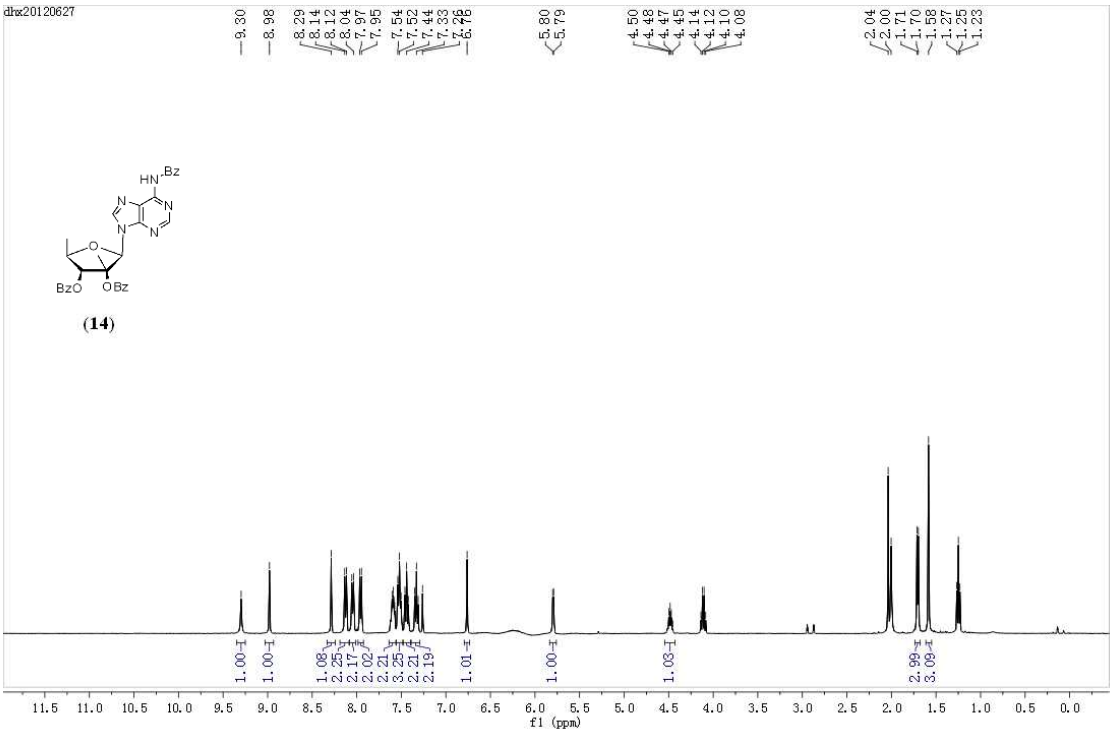
dhx20120627
9.30
8.98
8.29 8.14 8.12 8.04 7.97 7.95
7.54 7.52 7.44 7.33 7.26 6.76
5.80 5.79
4.50 4.48 4.47 4.45 4.14 4.12 4.10 4.08
2.04 2.00 1.71 1.70 1.58 1.27 1.25 1.23
1.00 1.00 1.08 2.25 2.17 2.02 2.21 3.25 2.21 2.19 1.01 1.00 1.03 2.99 3.09
HN Bz
N N N N O
BzO OBz
f1 (ppm)

(**14**)

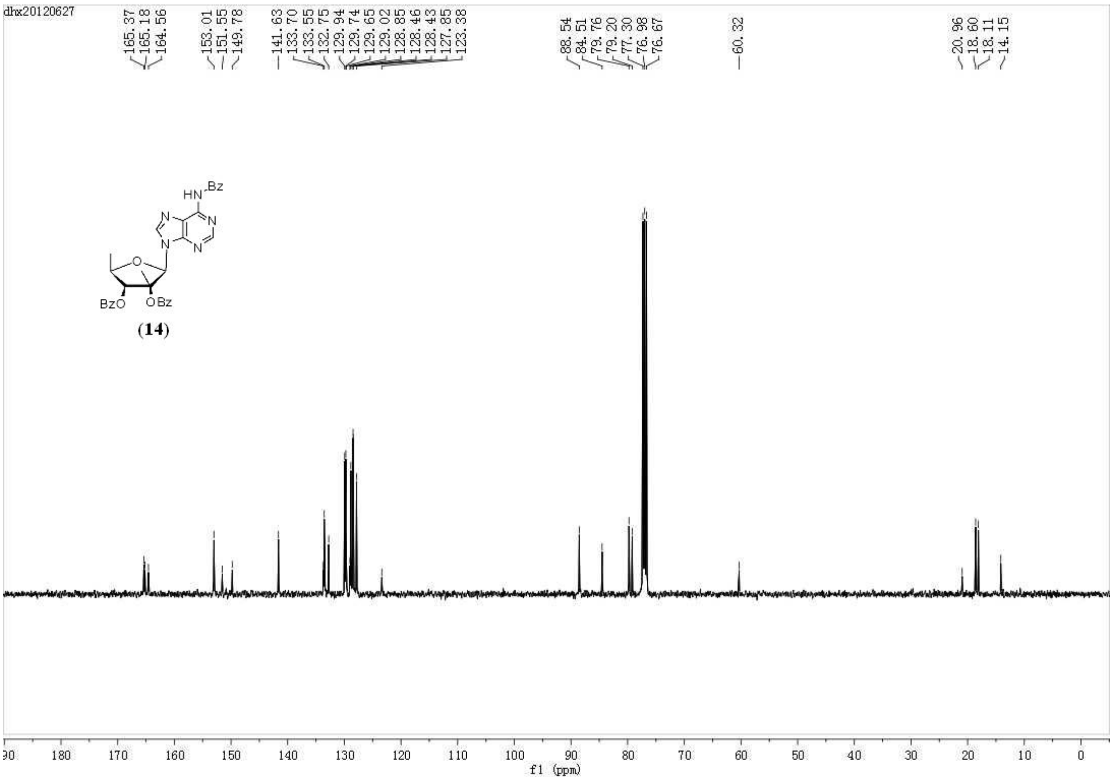
dhx20120627
165.37 165.18 164.56
153.01 151.55 149.78
141.63 133.70 133.55 132.75 129.94 129.74 129.65 129.02 128.85 128.46 128.43 127.85 123.38
88.54 84.51 79.76 79.20 77.30 76.98 76.67
60.32
20.96 18.60 18.11 14.15
HN Bz
N N N N O
BzO OBz
f1 (ppm)

(**14**)

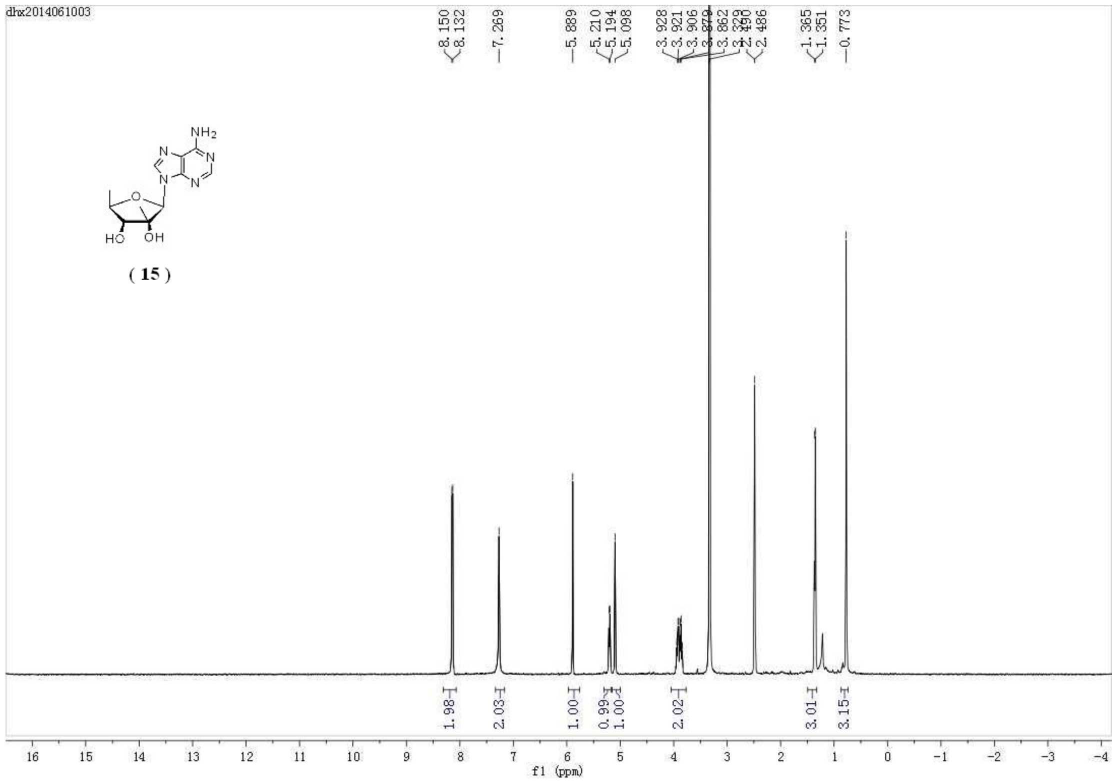

dhx2014061003
8.150
8.132
7.269
5.889
5.210
5.194
5.098
3.928
3.921
3.906
3.862
2.486
1.365
1.351
0.773
NH2
N
O
HO
OH
( 15 )
1.98
2.03
1.00
0.99
1.00
2.02
3.01
3.15
f1 (ppm)


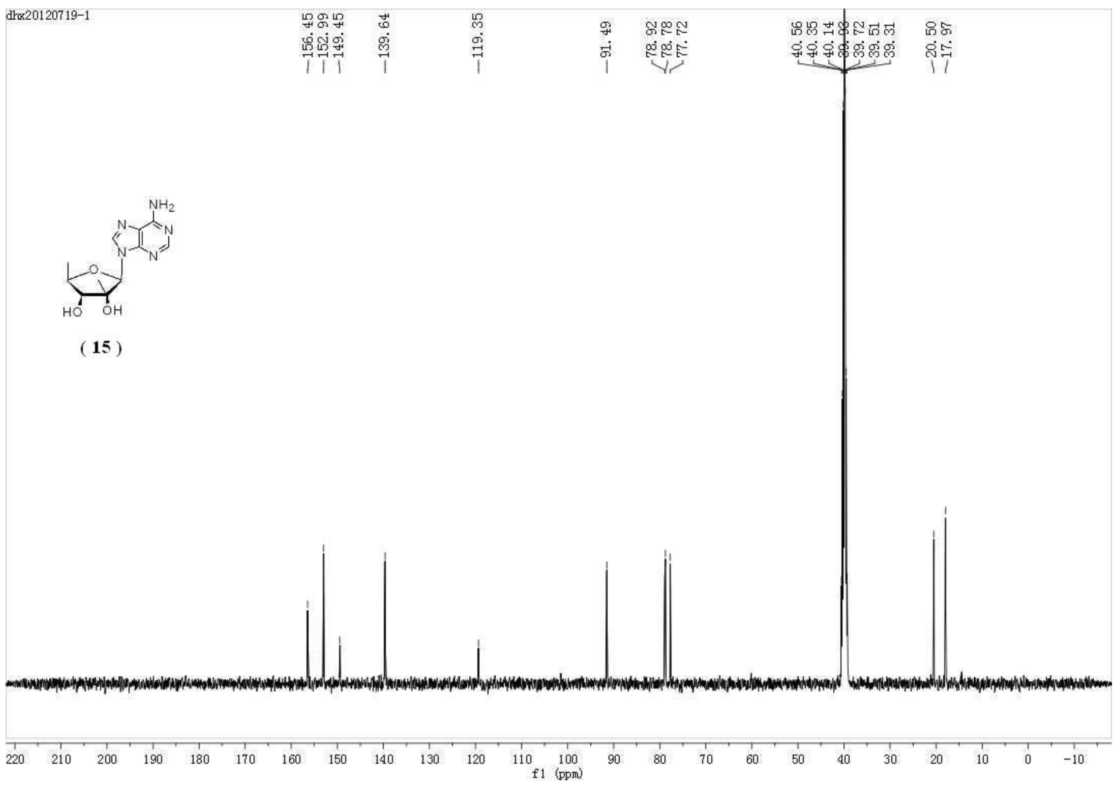

dhx20120719-1
156.45
152.99
149.45
139.64
119.35
91.49
78.92
78.78
77.72
40.56
40.35
40.14
39.72
39.51
39.31
20.50
17.97
NH2
N
O
HO
OH
( 15 )
f1 (ppm)

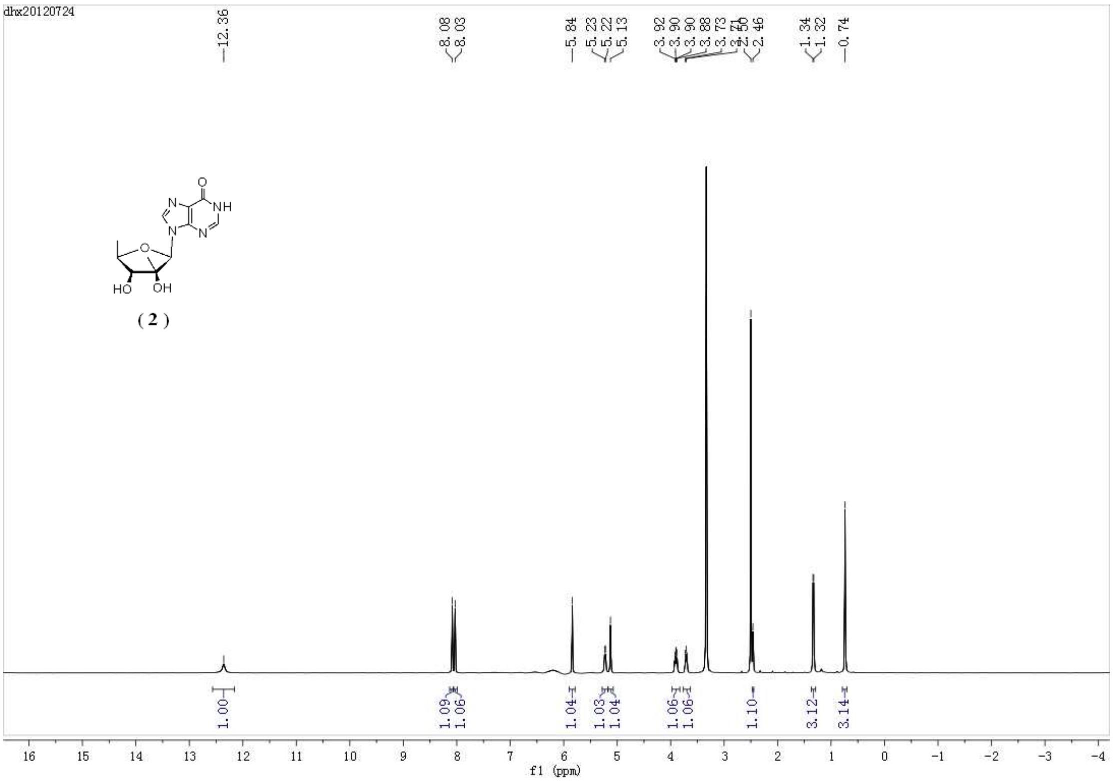
dhx20120724
12.36
8.08
8.03
5.84
5.23
5.22
5.13
3.92
3.90
3.90
3.88
3.73
3.71
2.50
2.46
1.34
1.32
0.74
NH
O
N
N
N
O
HO
OH
( 2 )
1.00
1.09
1.06
1.04
1.03
1.04
1.06
1.06
1.10
3.12
3.14
f1 (ppm)

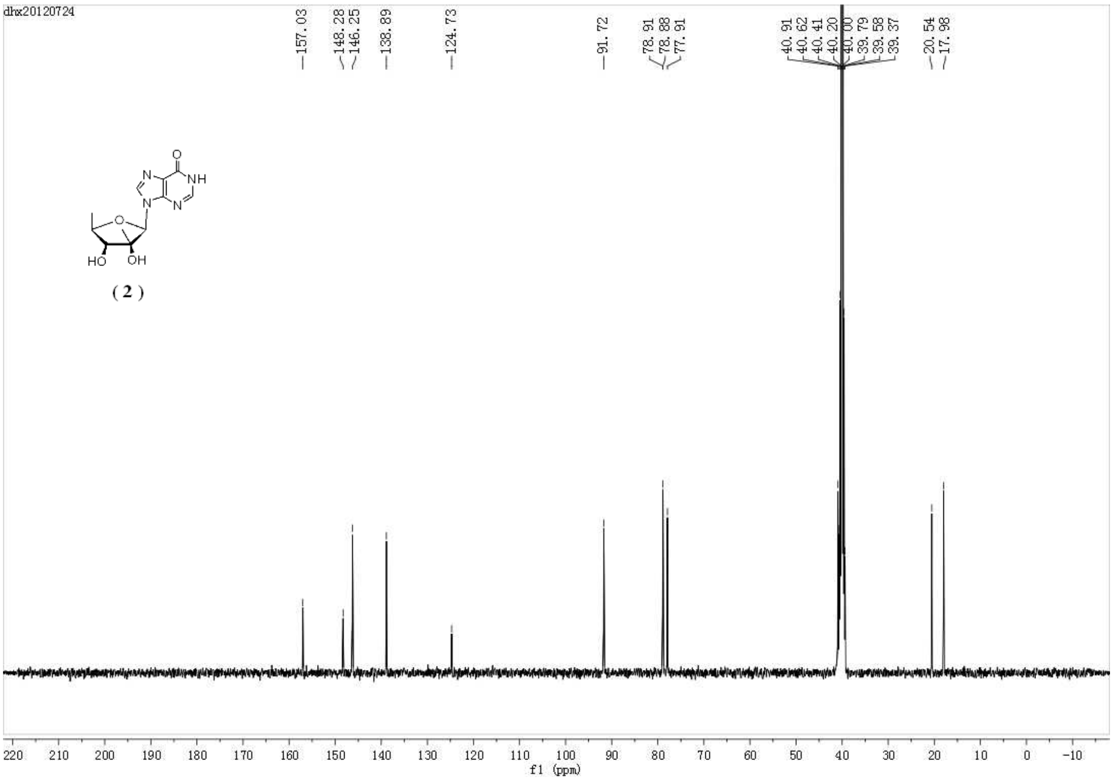
dhx20120724
157.03
148.28
146.25
138.89
124.73
91.72
78.91
78.88
77.91
40.91
40.62
40.41
40.20
40.00
39.79
39.58
39.37
20.54
17.98
NH
O
N
N
N
O
HO
OH
( 2 )
f1 (ppm)

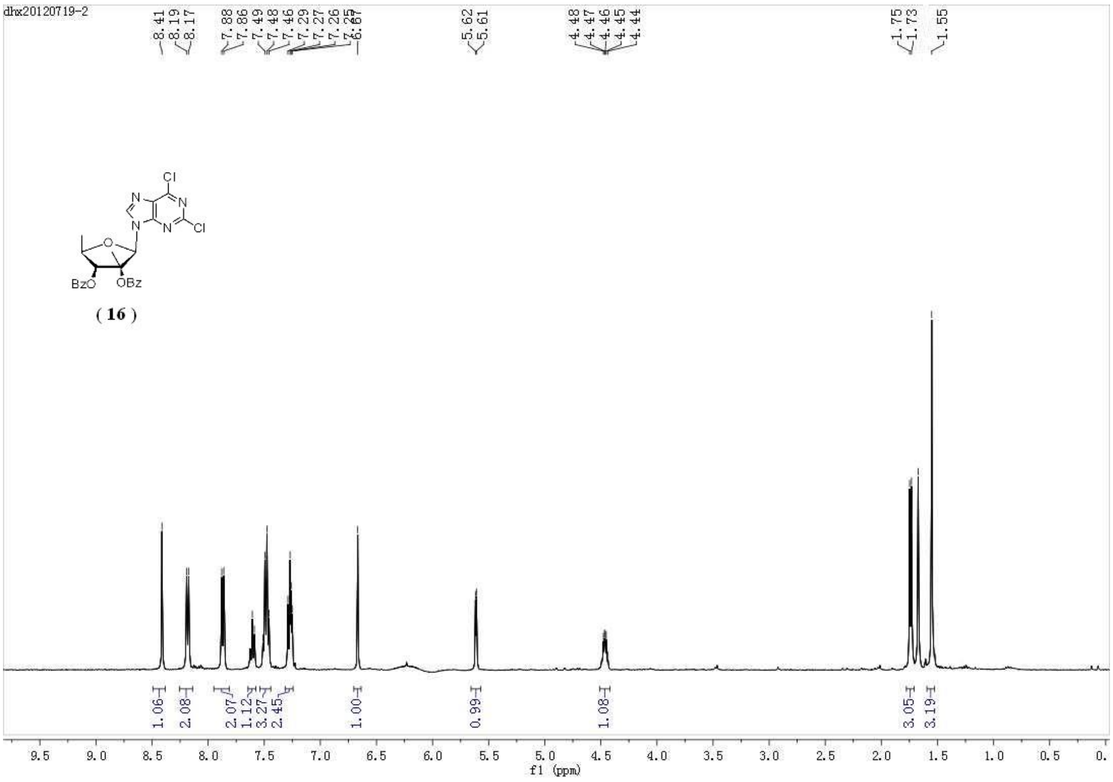
dhx20120719-2
8.41
8.19
8.17
7.88
7.86
7.49
7.48
7.46
7.29
7.27
7.26
7.25
6.67
5.62
5.61
4.48
4.47
4.46
4.45
4.44
1.75
1.73
1.55
Cl
N
N
N
N
Cl
O
BzO
OBz
( 16 )
1.06
2.08
2.07
1.12
3.27
2.45
1.00
0.99
1.08
3.05
3.19
f1 (ppm)

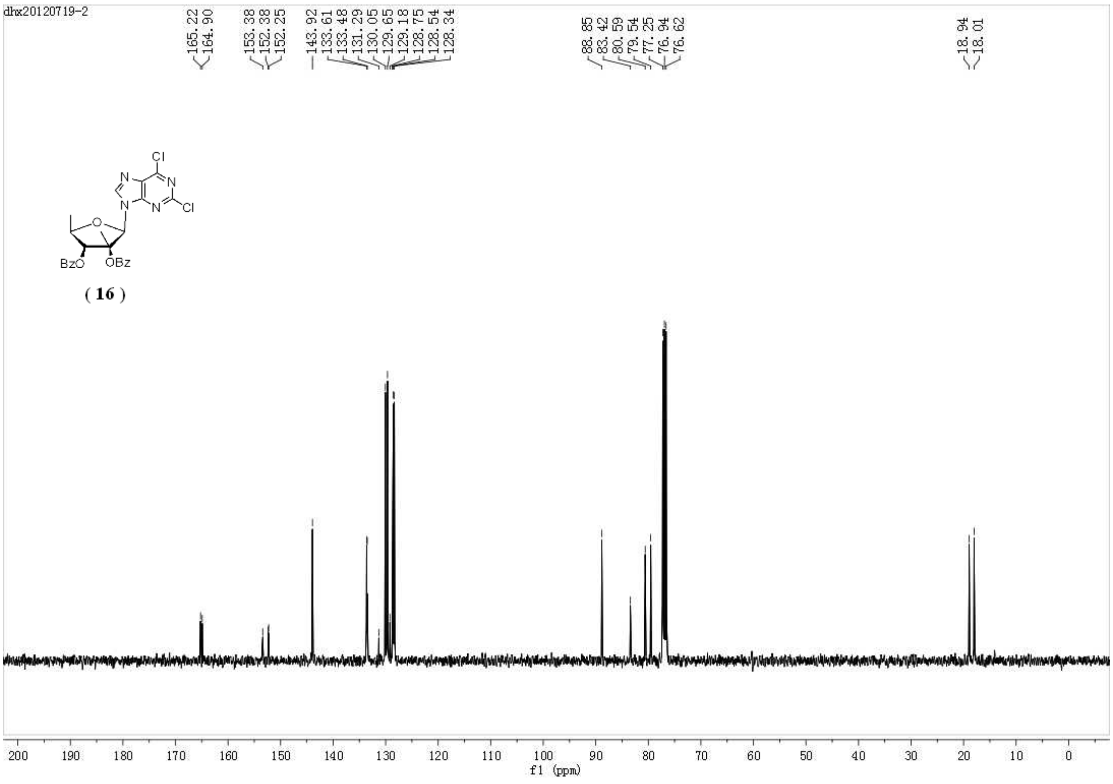
dhx20120719-2
165.22
164.90
153.38
152.38
152.25
143.92
133.61
133.48
131.29
130.05
129.65
129.18
128.75
128.54
128.34
88.85
83.42
80.59
79.54
77.25
76.94
76.62
18.94
18.01
Cl
N
N
N
N
Cl
O
BzO
OBz
( 16 )
f1 (ppm)

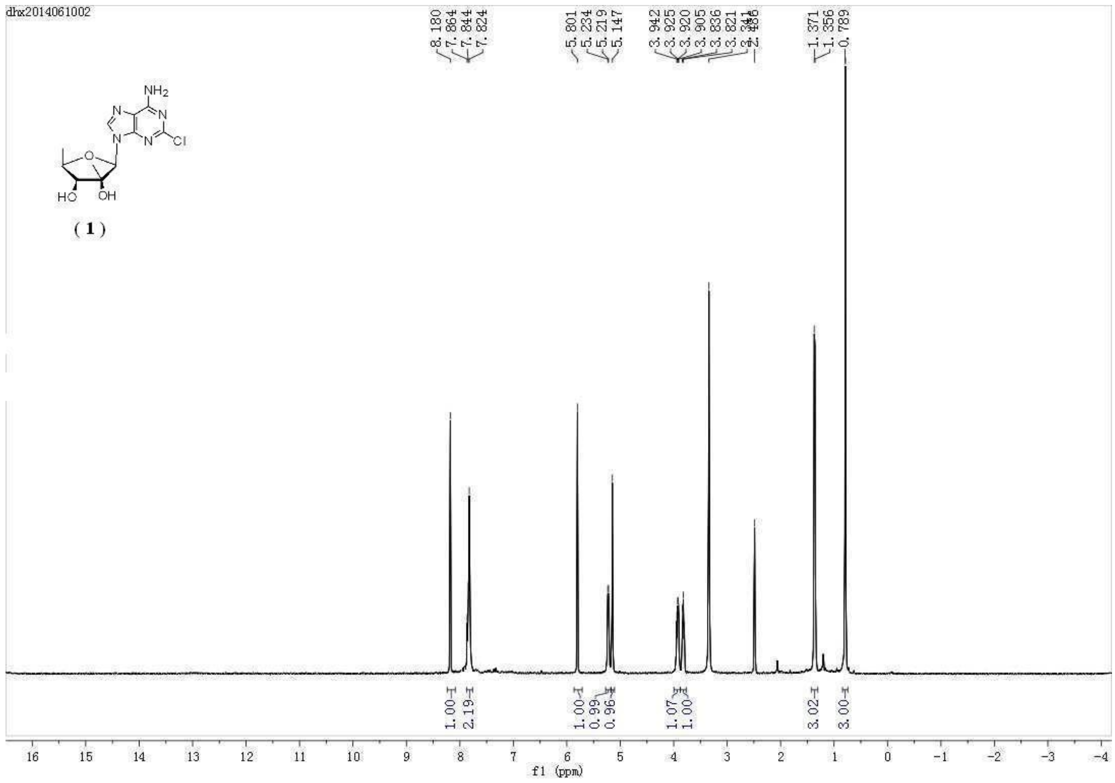

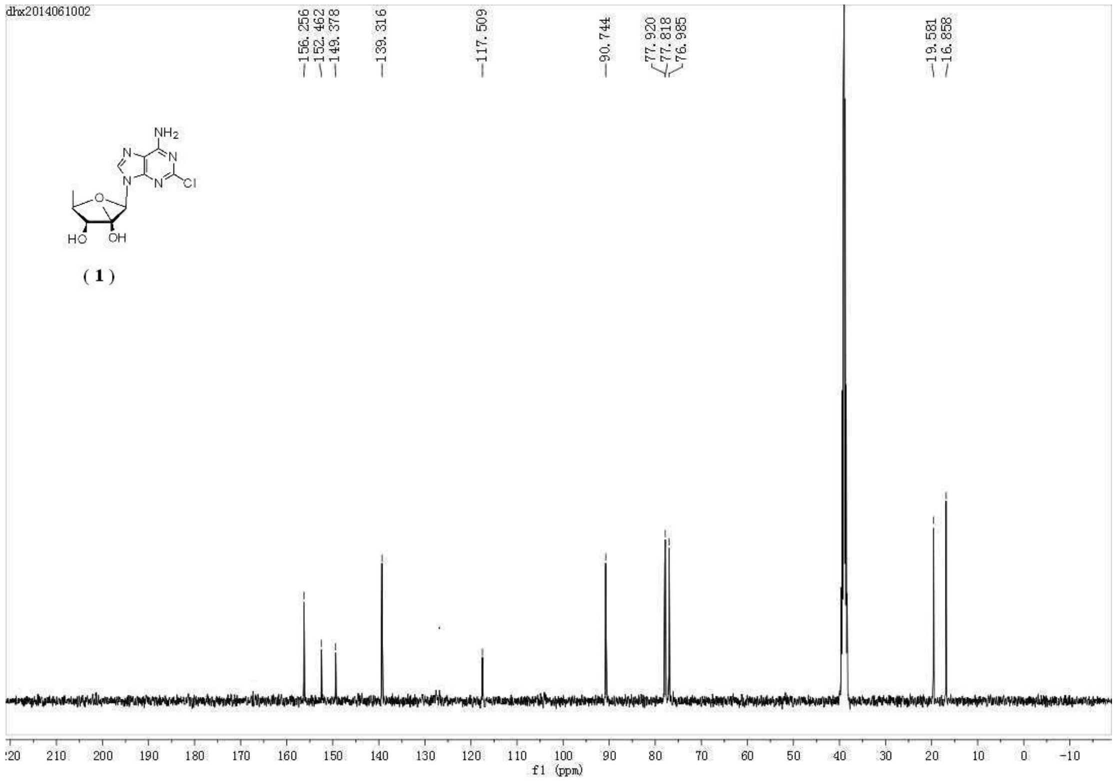

Supplement: File 1 — Detailed experimental procedures, characterization data of compounds, and copies of 1H and 13C NMR spectra. [file Beilstein_J_Org_Chem-10-1681-s001.pdf]
